# Supplementary material for: Lipid Traffic Analysis reveals the impact of high paternal carbohydrate intake on offsprings’ lipid metabolism
Source: Commun Biol. 2021 Feb 5;4:163. doi: 10.1038/s42003-021-01686-1 (PMC7864968; doi:10.1038/s42003-021-01686-1)
Supplement: Supplementary file 2 — Supplementary Information [file 42003_2021_1686_MOESM2_ESM.pdf]

Supplementary Information

Furse *et al.*, Lipid traffic analysis reveals the impact of high paternal carbohydrate intake on offsprings' lipid metabolism.

F1A Liv-Ser NP-NC	F1A Ser-Ceb NP-NC	F1A Ser-RiB NP-NC	F1A Ser-Hea NP-NC	F1A Ser-Adi NP-NC
TG(33:00)[ø]	TG(33:00)[ø]	TG(33:00)[ø]	TG(33:00)[ø]	
	TG(35:01)[ø]	TG(35:01)[ø]		
TG(48:01)[ø]			TG(48:01)[ø]	
TG(50:02)[ø]			TG(50:02)[ø]	TG(50:02)[ø]
TG(50:03)[ø]			TG(50:03)[ø]	TG(50:03)[ø]
TG(52:01)[ø]			TG(52:01)[ø]	TG(52:01)[ø]
TG(52:02)[ø]	TG(52:02)[ø]		TG(52:02)[ø]	TG(52:02)[ø]
TG(52:03)[ø]	TG(52:03)[ø]		TG(52:03)[ø]	TG(52:03)[ø]
TG(52:05)[ø]	TG(52:05)[ø]		TG(52:05)[ø]	TG(52:05)[ø]
TG(54:02)[ø]			TG(54:02)[ø]	
TG(54:03)[ø]			TG(54:03)[ø]	TG(54:03)[ø]
TG(54:04)[ø]			TG(54:04)[ø]	TG(54:04)[ø]
TG(54:05)[ø]			TG(54:05)[ø]	TG(54:05)[ø]
TG(54:06)[ø]			TG(54:06)[ø]	TG(54:06)[ø]
TG(54:07)[ø]			TG(54:07)[ø]	
TG(56:05)[ø]			TG(56:05)[ø]	TG(56:05)[ø]
TG(56:06)[ø]			TG(56:06)[ø]	
TG(56:07)[ø]			TG(56:07)[ø]	
TG(56:08)[ø]			TG(56:08)[ø]	
	DG(20:00)[§]			
DG(26:00)[§]			DG(26:00)[§]	
		DG(31:02)[§]		
DG(32:03)[§]			DG(32:03)[§]	
DG(33:01)[§]			DG(33:01)[§]	DG(33:01)[§]
DG(34:02)[ø]	DG(34:02)[ø]	DG(34:02)[ø]	DG(34:02)[ø]	
DG(34:03)[§]			DG(34:03)[§]	DG(34:03)[§]
		DG(36:01)[§]		
DG(36:05)[§]			DG(36:05)[§]	
DG(37:04)[§]		DG(37:04)[§]		
DG(40:04)[§]	DG(40:04)[§]	DG(40:04)[§]	DG(40:04)[§]	
DG(40:07)[§]	DG(40:07)[§]	DG(40:07)[§]	DG(40:07)[§]	
DG(42:04)[§]	DG(42:04)[§]		DG(42:04)[§]	
	DG(44:07)[§]	DG(44:07)[§]	DG(44:07)[§]	DG(44:07)[§]
MG(20:03) [ø]	MG(20:03) [ø]	MG(20:03) [ø]	MG(20:03) [ø]	

Table S1. Triglyceride variables on the Liver-Serum (Liv-Ser) axis in the control (NP-NC) group that also appear on the Serum-Cerebellum (Ser-Ceb), Serum-Right Brain (Ser-RiB), Serum-Heart (Ser-Hea) or Serum-Adipose (Ser-Adi) axes of the control group (NP-NC). [ø]Ammoniated adduct, [§]Protonated, water-loss ion; *Sodiated adduct. DG, diglyceride (water-loss adduct from fragmentation in source); TG, triglyceride.

Liv-Ser LP-HC	Ser-Adi NP-NC	Ser-CEB LP-HC	Ser-RiB LP-HC	Ser-Hea LP-HC
	DG(33:01)§	DG(33:01)§	DG(33:01)§	
DG(35:01)§		DG(35:01)§	DG(35:01)§	DG(35:01)§
TG(50:02)ø		TG(50:02)ø	TG(50:02)ø	
TG(50:03)ø	TG(50:03)ø			
TG(52:01)ø	TG(52:01)ø			
TG(52:02)ø			TG(52:02)ø	
	TG(52:05)ø		TG(52:05)ø	
TG(52:03)ø			TG(52:03)ø	TG(52:03)ø
TG(54:03)ø	TG(54:03)ø			
TG(54:04)ø			TG(54:04)ø	
TG(58:08)ø				TG(58:08)ø
TG(58:09)ø				TG(58:09)ø

Table S2. Triglyceride variables on the Liver-Serum (Liv-Ser) axis in the control (NP-NC) group of F1As that also appear on the Serum-Cerebellum (Ser-Ceb), Serum-Right Brain (Ser-RiB), Serum-Heart (Ser-Hea) or Serum-Adipose (Ser-Adi) axes of the control group. §Protonated, water-loss ion; *Sodiated adduct; øAmmoniated adduct. DG, diglyceride (water-loss adduct from fragmentation in source); TG, triglyceride.

F2N CEB NP-NC
TG(37:00)§
TG(43:02)§
TG(43:03)*
TG(44:03)*
TG(45:03)§
TG(46:01)§
TG(47:04)§
TG(49:01)§
TG(49:03)§
TG(49:06)§
TG(54:11)§
DG(37:03)*
DG(38:04)*
DG(40:01)ø
DG(40:02)ø
DG(41:00)ø
DG(42:01)ø
DG(42:07)ø
DG(42:07)*
DG(43:05)*

Table S3. Triglyceride variables unique to the cerebella of normal protein-normal carbohydrate (NP-NC) F2N individuals. §Sodiated adduct; *Ammoniated adduct; øProtonated, water-loss ion. CEB, cerebellum; DG, diglyceride (water-loss adduct from fragmentation in source); TG, triglyceride.

F2N Liv-Ser NP-NC	F2N Ser-Ceb NP-NC	F2N Ser-Hea NP-NC
		PC(30:3)*
		PC(36:3)*
		PC(38:5)*
	PC(39:2)*	
PC(39:3)§	PC(39:3)§	
	PC(39:4)§	
		PC(39:7)*
		PC(39:8)§
PC(40:4)§	PC(40:4)§	
PC(40:6)§	PC(40:6)§	PC(40:6)§
		PC(40:7)*
PC(41:4)§	PC(41:4)§	
PC(42:4)§		
PC(44:2)*		
PC(46:2)*		

Table S4. Phosphatidylcholine variables on the Liver-Serum (Liv-Ser) axis in the control (NP-NC) group of F2Ns that are also found on the Serum-Cerebellum (Ser-Ceb) and/or Serum-Heart (Ser-Hea) axis of the same (NP-NC) group. §Chloride adduct; *acetate adduct. PC, phosphatidylcholine.

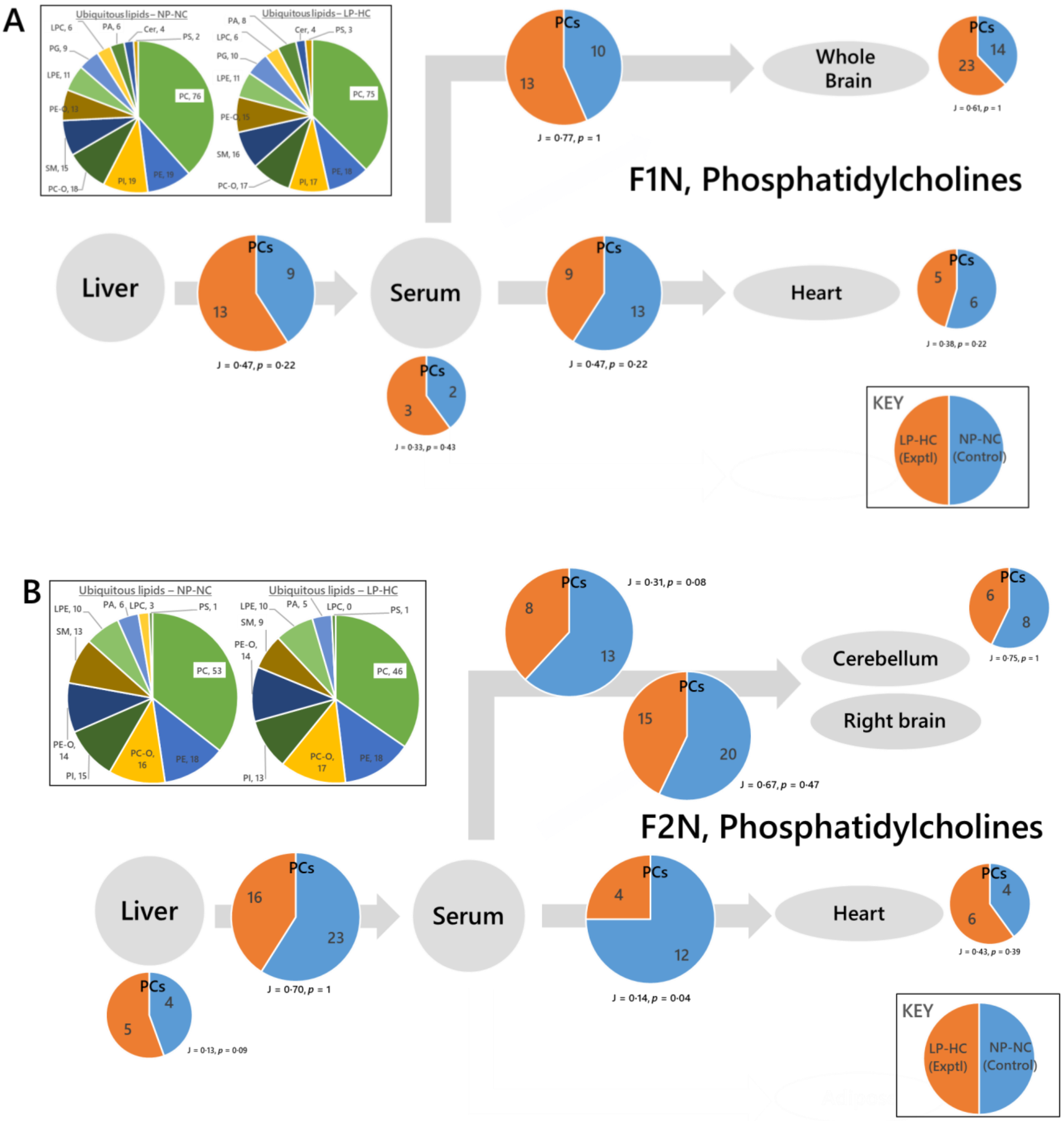

Fig. S1. Switch analyses of phosphatidylcholines in control (NP-NC) mice and a phenotype whose (grand)sires were fed a low-protein, high carbohydrate (LP-HC) diet, measured by mass spectrometry in negative ionisation mode. Panel **A**, F1 Neonates (F1N); **B**, F2 Neonates (F2N). The pie charts in the insert show the number of ubiquitous lipid variables for that network, for each phenotype. Larger pie charts with *J* values represent PC variables found in two adjacent compartments. Smaller pie charts with *J* values represent PC variables found in isolated in compartments. *J* represents the Jaccard-Tanimoto coefficient for the comparison, with accompanying *p* value, as a measure of the similarity between the variables identified in the two phenotypes for each comparison. The *p* value shown represents the probability that the difference between the lists of variables for the two phenotypes occurred by random chance. Orange is used for the LP-HC group whereas blue is used for the NP-NC group. LPC, *lyso*-phosphatidylcholine; LPE *lyso*-phosphatidylethanolamine; PA, phosphatidic acid; PC, phosphatidylcholine; PC-O, phosphatidylcholine plasmalogen; PE, phosphatidylethanolamine; PE-O, phosphatidylethanolamine plasmalogen; PG, phosphatidylglycerol; PI, phosphatidylinositol; PS, phosphatidylserine; SM, sphingomyelin; TG, triglyceride.

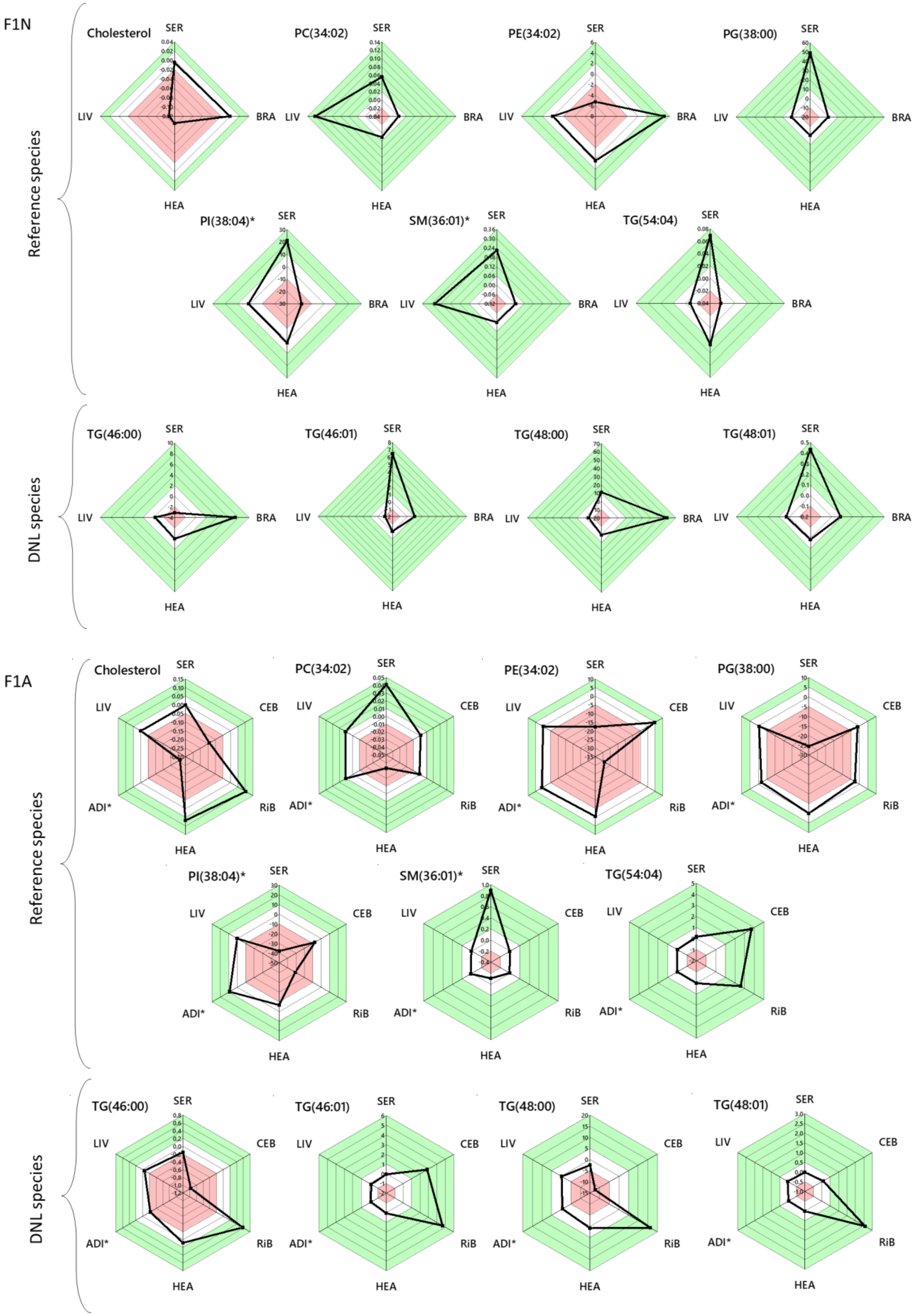
F1N
Reference species
Cholesterol
PC(34:02)
PE(34:02)
PG(38:00)
PI(38:04)*
SM(36:01)*
TG(54:04)
DNL species
TG(46:00)
TG(46:01)
TG(48:00)
TG(48:01)
SER
BRA
HEA
LIV
F1A
Reference species
Cholesterol
PC(34:02)
PE(34:02)
PG(38:00)
PI(38:04)*
SM(36:01)*
TG(54:04)
DNL species
TG(46:00)
TG(46:01)
TG(48:00)
TG(48:01)
SER
CEB
RiB
HEA
ADI*
LIV

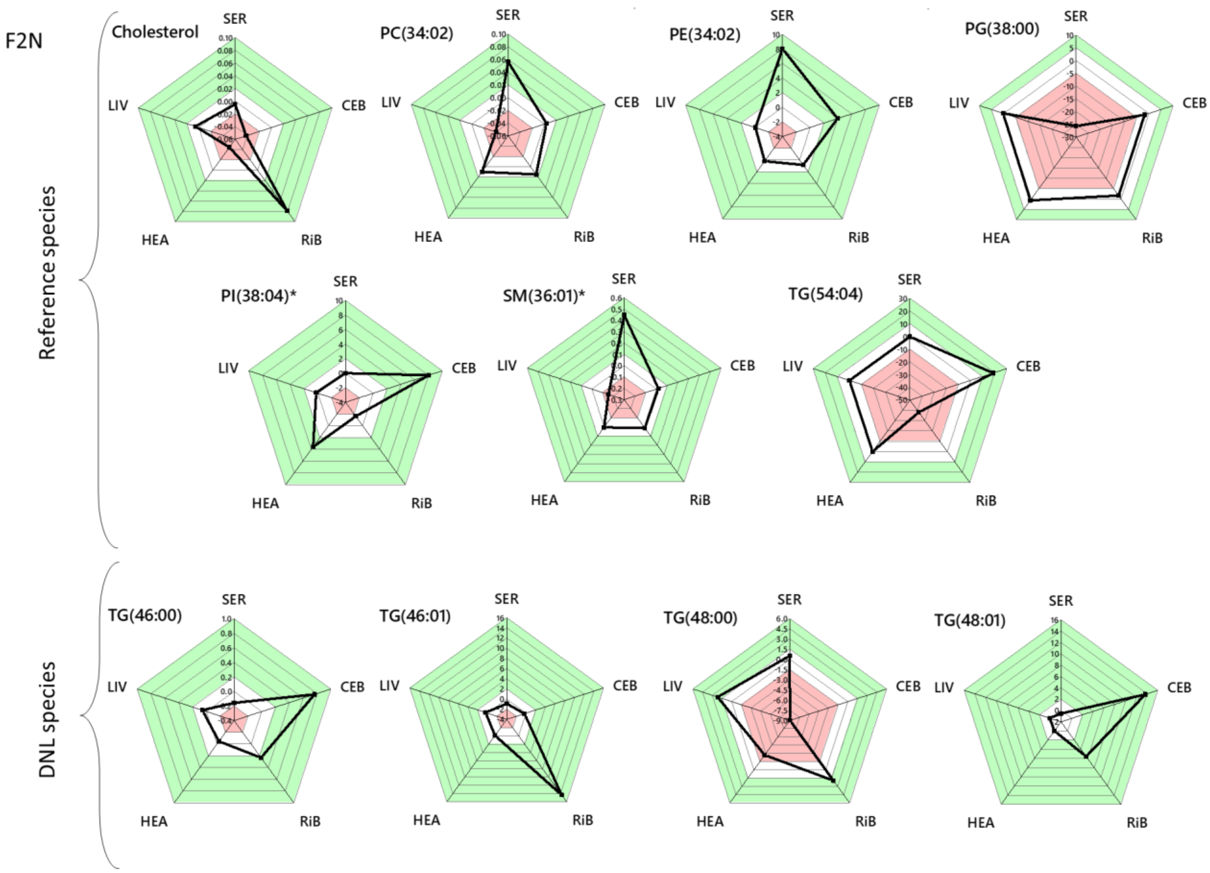

Fig. S2. Radar plots of the fold change in abundance of lipid variables (error-normalised) unrelated (rows 1 and 2) and related (row 3) to de novo lipogenesis in mice. Panel **A**, F1 Neonate compartments; **B**, F1 adult compartments; **C**, F2 neonate compartments. The value given is the log of the mean of experimental abundance values divided by the mean of control values, divided by the propagated error for that variable (Eq. 1). Values of 0 show no change between phenotypes, negative values show lower in the experimental group (LP-HC offspring), positive values show increased abundance in the LP-HC offspring. The white areas represent the 0 point and one division above and below this. The red areas represent values more negative, and the green areas values more positive than this. ADI*, adipose (petrol wash); BRA, brain; CEB, cerebellum; HEA, heart; LIV, liver; RiB, right brain; SER, serum. PC, phosphatidylcholine; PE, phosphatidylethanolamine; PG, phosphatidylglycerol; PI, phosphatidylinositol; SM, sphingomyelin; TG, triglyceride.

Lipid Class	Isoform	Mass (M_r)	Stock concentration (µM)
Cholesteryl ester	CE(18:0-d_6)	658.6603	3.80
Ceramide	C16-d_{31} Ceramide	566.6928	4.40
Fatty acid	C15:0-d_{29} FA	271.4076	9.23
Fatty acid	C17:0-d_{33} FA	303.4635	8.25
Fatty acid	C20:0-d_{39} FA	351.5484	7.13
lyso-Phosphatidylcholine	*lyso*-PC(C14:0)-d_{42}	421.5481	5.93
Phosphatidic acid	PA(C16:0-d_{31}/C18:1) Na^+ salt	704.6784	3.55
Phosphatidylcholine	PC(C16:0-d_{31}/C18:1)	789.7637	3.18
Phosphatidylethanolamine	PE(C16:0-d_{31}/C18:1)	747.7171	3.35
Phosphatidylglycerol	PG(C16:0-d_{31}/C18:1) Na^+ salt	778.7157	3.20
Phosphatidylinositol	PI(C16:0-d_{31}/C18:1) NH_4^+ salt	881.7441	1.13
Phosphatidylserine	PS(C16:0-d_{62}) Na^+ salt	733.7794	3.40
Sphingomyelin	SM(C16:0-d_{31})	733.7603	2.04
Triglyceride	TG(45:0-d_{29})	852.2314	2.93
Triglyceride	TG(48:0-d_{31})	900.3167	2.78
Triglyceride	TG(54:0-d_{35})	996.4851	2.50

Table S5. List of internal standards used for lipid profiling in the present study.
