# Supplementary material for: Lipid Traffic Analysis reveals the impact of high paternal carbohydrate intake on offsprings’ lipid metabolism
Source: Commun Biol. 2021 Feb 5;4:163. doi: 10.1038/s42003-021-01686-1 (PMC7864968; doi:10.1038/s42003-021-01686-1)
Supplement: Supplementary file 3 — Description of Additional Supplementary Files [file 42003_2021_1686_MOESM3_ESM.pdf]

Description of Additional Supplementary Files

File Name: Supplementary Data 1

Description: Network diagrams showing which and how many lipid variables were detected across all compartments (pie charts, A type/ubiquitous lipids), between two tissues (blue tables, B type/adjacent lipids) and in only one compartment (orange tables, U type/unique lipids) in the two phenotypes studied. NP-NC, normal protein-normal carbohydrate; LP-HC, low protein, high carbohydrate. Cer, ceramide; Chol, cholesterol; LPC, lyso-phosphatidylcholine; LPE, lyso-phosphatidylethanolamine; LPG, lyso-phosphatidylglycerol; PA, phosphatidic acid; PC, phosphatidylcholine; PC-O, phosphatidylcholine plasmalogen; PE, phosphatidylethanolamine; PE-O, phosphatidylethanolamine plasmalogen; PG, phosphatidylglycerol; PI, phosphatidylinositol; PS, phosphatidylserine; S & SE, Sterols and Steryl Esters; SM, sphingomyelin; TG, triglyceride (comprises diglyceride water-loss adducts from fragmentation in source).

File Name: Supplementary Data 2

Description: All 31P NMR data generated from pooled samples used in the present study.

File Name: Supplementary Data 3

Description: The R code used to produce the Switch and Abundance analyses used in the present study

File Name: Supplementary Data 4

Description: All Abundance analyses for the comparisons in the present study.

File Name: Supplementary Data 5

Description: Annotations (with appropriate isobars) of signals identified in the present study using DI-MS.
