# Supplementary material for: Lipid Traffic Analysis reveals the impact of high paternal carbohydrate intake on offsprings’ lipid metabolism
Source: Commun Biol. 2021 Feb 5;4:163. doi: 10.1038/s42003-021-01686-1 (PMC7864968; doi:10.1038/s42003-021-01686-1)
Supplement: Supplementary file 4 — Supplementary Data 1 [file 42003_2021_1686_MOESM4_ESM.pdf]

Lipid traffic analysis reveals the impact of high paternal carbohydrate intake on offsprings' lipid metabolism

Supplementary data 1

Furse *et al.*

Switch Analyses
(all variables)

Sections

- F1 neonates, positive ionisation mode
- F1 adults, positive ionisation mode
- F2 neonates, positive ionisation mode
- F1 neonates, negative ionisation mode
- F1 adults, negative ionisation mode
- F2 neonates, negative ionisation mode

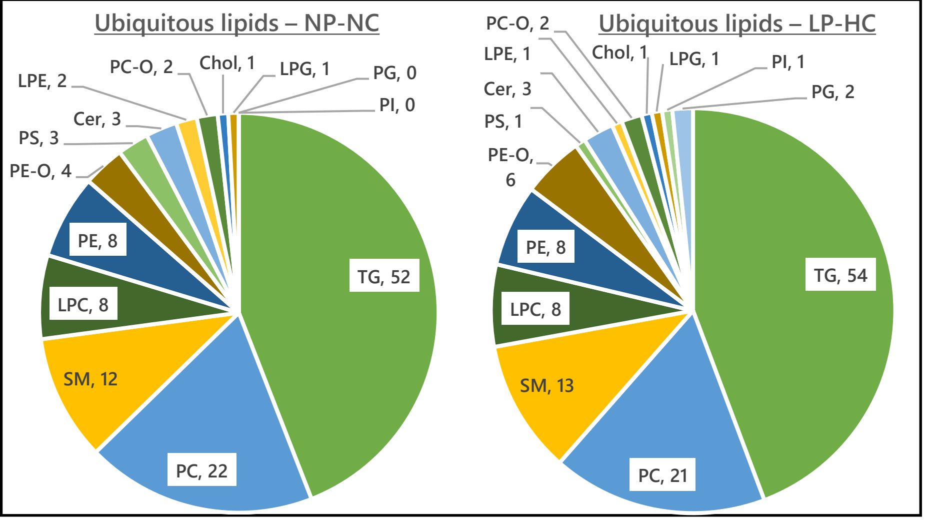

	J	p
TG	0·96	0·011
PC	0·95	1
SM	0·92	1
LPC	1	1
PE	1	1
PE-O	0·67	1
PS	0·33	1
LPE	0·50	1
LPG	1	1
PC-O	1	1
PG	0	1
PI	0	1
Cer	1	1
Chol	1	1

F1N, +ve

Liver

U_{LIV}

	NP-NC	LP-HC	J	p
TG	2	0	0	1
PI	1	1	0	0·25

$B_{LIV\text{-}SER}$

	NP-NC	LP-HC	J	p
TG	12	13	0·39	0·08
LPE	3	2	0·33	1
PG	3	2	0	0·17
PC	2	2	0·33	0·44
PI	2	2	0·33	0·44
PS	2	2	0·33	0·44
Cer	1	2	0	0·15
LPC	0	1	0	1
PC-O	0	1	0	1
PE	0	3	0	1
PE-O	0	4	0	1
SM	0	3	0	1

Serum

U_{SER}

	NP-NC	LP-HC	J	p
TG	8	4	0·09	0·04
PI	6	0	0	1
PS	6	3	0	0·03
S & SE	3	4	0·4	0·36
LPC	2	2	1	1
Cer	1	2	0	0·15
PA	1	0	0	1
PG	1	0	0	1
SM	1	1	0	0·25

$B_{SER\text{-}BRA}$

	NP-NC	LP-HC	J	p
TG	48	65	0·59	0·19
PS	15	13	0·60	0·22
PI	14	20	0·45	0·13
PG	13	15	0·87	1
Cer	12	17	0·53	0·26
S & SE	7	9	0·75	0·39
PC-O	7	8	0·63	0·48
LPG	3	3	1	1
LPI	3	4	0·75	1
LPC	5	5	1	1
PC	5	7	0·50	0·40
PE	5	7	0·67	1
LPE	5	5	1	1
SM	4	8	0·43	1
PE-O	2	9	0·22	1
PA	1	2	0	1

Whole Brian

U_{BRA}

	NP-NC	LP-HC	J	p
TG	33	30	0·44	0·22
PI	15	13	0·55	0·23
PS	13	16	0·53	0·20
PC-O	11	14	0·56	0·31
Cer	11	8	0·11	0·36
PC	10	10	1	1
PG	9	10	0·46	0·19
PI-O	3	8	0·10	0·05
SM	6	5	0·37	0·22
PE-O	5	4	0·80	1
PE	4	4	0·33	0·24
PA	1	1	1	1
LPS	1	1	1	1
S & SE	0	3	0	1

$B_{SER\text{-}HEA}$

	NP-NC	LP-HC	J	p
TG	16	18	0·70	0·45
PS	4	1	0·25	1
PC-O	3	3	0·50	0·43
SM	3	3	0·50	0·43
PI	2	1	0	0·15
LPO	2	1	0·50	1
PC	2	0	0	1
PE	2	3	0·25	0·43
LPE	2	0	0	1
PG	1	3	0·33	1
LPC	1	0	0	1
PE-O	1	5	0·20	1
CE	0	2	0	1

Heart

U_{HEA}

	NP-NC	LP-HC	J	p
TG	6	4	0·25	0·15
PC-O	2	0	0	1

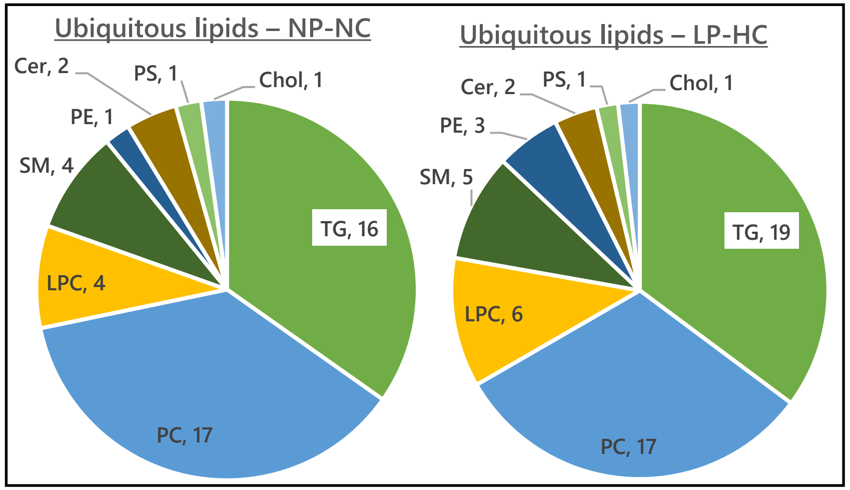

	J	*p*
TG	0·84	1
PC	0·89	0·41
LPC	0·67	1
SM	0·80	1
PE	1	3
Cer	2	2
PS	1	1
Chol	1	1
PA	0·33	1

F1A, +ve

Liver

Serum

Cerebellum

Right brain

Heart

Adipose

$B_{LIV-SER}$

	NP-NC	LP-HC	*J*	*p*
TG	29	36	0·80	0·36
PE-O	4	4	1	1
SM	4	6	0·67	1
PE	3	5	0·60	1
LPC	3	4	0·75	1
PC	2	2	1	1
LPG	2	1	0·50	1
Cer	1	1	1	1
LPE	0	2	0	1

U_{LIV}

	NP-NC	LP-HC	*J*	*p*
TG	4	2	0·20	0·22
PI	1	3	0·33	1
SE	0	1	0	1
SM	1	0	0	1
PS	1	1	1	1

U_{SER}

	NP-NC	LP-HC	*J*	*p*
S & SE	6	6	1	1
LPC	4	3	0·75	1
LPE	1	1	1	1
PC	1	0	0	1
LPI	0	1	0	1
PG	1	0	0	1
SM	1	1	1	1

$B_{SER-CEB}$

	NP-NC	LP-HC	*J*	*p*
TG	12	18	0·67	1
PE-O	5	4	0·80	1
SM	1	3	0·33	1
PE	5	6	0·83	1
LPC	4	5	0·80	1
PC	3	2	0·67	1
LPG	3	1	0·33	1
Cer	2	2	1	1
LPE	2	3	0·67	1
SE	1	1	1	1
LPI	1	0	0	1
PS	1	0	0	1

$B_{SER-RiB}$

	NP-NC	LP-HC	*J*	*p*
TG	10	20	0·50	0·16
LPC	4	5	0·80	1
PE-O	5	4	0·80	1
PE	3	4	0·75	1
LPE	2	3	0·67	1
PC	3	2	0·67	1
LPG	3	2	0·67	1
Cer	2	2	1	1
SM	1	2	0·50	1
SE	1	1	1	1
PS	1	0	0	1

U_{CEB}

	NP-NC	LP-HC	*J*	*p*
TG	8	6	0·53	0·38
PI	2	4	0·50	1
PC	1	4	0·25	1
PE	2	2	0·33	0·44
PS	1	2	0·50	1
SM	2	1	0·50	1
Cer	1	1	0	0·33
PC-O	1	1	1	1

U_{RiB}

	NP-NC	LP-HC	*J*	*p*
TG	0	2	0	1
PI	1	3	0·33	1
PS	1	2	0·50	1
SM	0	1	0	1
PC	1	0	0	1
PE	1	0	0	1
SE	1	0	0	1
PG	1	0	0	1

$B_{SER-HEA}$

	NP-NC	LP-HC	*J*	*p*
TG	29	34	0·85	0·47
PE-O	5	4	0·80	1
LPC	4	5	0·80	1
PE	4	4	0·60	0·43
SM	3	5	0·60	1
PC	3	2	0·67	1
LPE	2	3	0·67	1
LPG	1	2	0·50	1
PS	1	0	0	1
Cer	0	1	0	1

U_{HEA}

	NP-NC	LP-HC	*J*	*p*
TG	12	14	0·37	0·06
PI	1	0	0	1
PS	1	1	1	1
SM	2	0	0	1
PC	0	1	0	1
PG	0	1	0	1
PC-O	1	0	0	1

$B_{SER-ADI}$

	NP-NC	LP-HC	*J*	*p*
TG	14	7	0·50	1
LPE	2	1	0·50	1
SM	2	1	0	0·29
PE	1	3	0·33	1
LPC	0	2	0	1
PC	1	1	1	0·13

U_{ADI}

	NP-NC	LP-HC	*J*	*p*
TG	2	0	0	1
PI	2	1	0·50	1
PS	2	0	0	1
SM	1	1	1	1

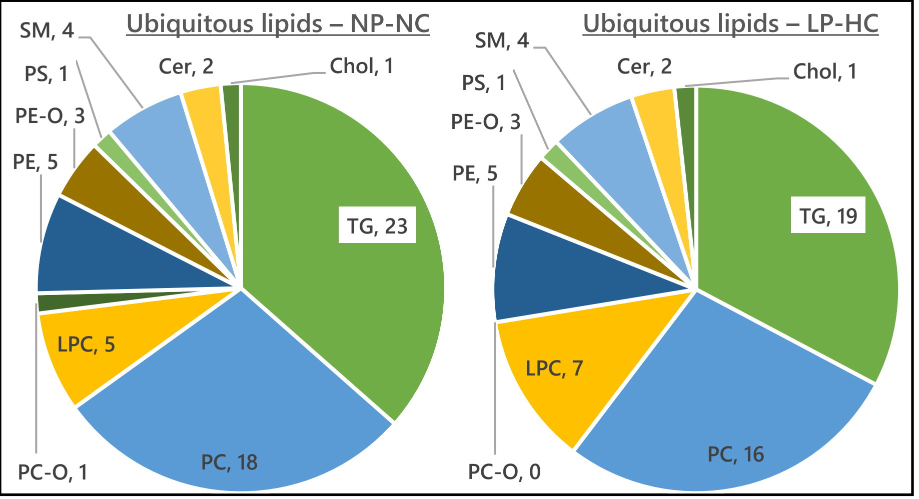

	J	p
TG	0·83	1
PC	0·95	0·03
LPC	0·71	1
PC-O	0	1
PE	0·67	0·43
PE-O	1	1
PS	1	1
SM	1	1
Cer	1	1
Chol	1	1

F2N +ve

Liver

U_{LIV}

	NP-NC	LP-HC	J	p
TG	1	2	0	0·15
PI	1	0	0	1
SM	1	0	0	1
CE	1	0	0	1

$B_{LIV-SER}$

	NP-NC	LP-HC	J	p
TG	26	27	0·83	0·47
PC	2	1	0·5	1
LPC	2	3	0·67	1
PC-O	1	0	0	1
PE	1	1	0	0·25
LPE	2	1	0·5	1
PE-O	1	1	1	1
LPG	3	2	0·67	1
SM	3	2	0·67	1

Serum

U_{SER}

	NP-NC	LP-HC	J	p
TG	2	3	0	0·13
S & SE	3	3	1	1
LPC	5	3	0·6	1
SM	0	1	0	1

$B_{SER-CEB}$

	NP-NC	LP-HC	J	p
TG	19	12	0·48	0·21
PC	2	1	0	0·14
LPC	4	3	0·75	1
PC-O	1	0	0	1
PE	2	1	0	0·14
LPE	3	3	1	1
PE-O	4	4	1	1
LPG	3	2	0·67	1
SM	2	0	0	1
PG	1	1	1	1
PI	2	1	0·5	1
PS	1	1	1	1
S & SE	1	1	1	1
Cer	2	1	0·5	1
LPI	1	1	1	1

$B_{SER-RiB}$

	NP-NC	LP-HC	J	p
TG	10	11	0·62	0·40
PC	3	2	0·25	0·43
LPC	1	3	0·33	1
PC-O	1	1	0	1
PE	1	1	0	1
LPE	3	3	1	1
PE-O	1	1	1	1
PG	1	1	1	1
LPG	3	3	1	1
Cer	2	2	1	1
LPI	1	1	1	1
PI	2	1	0·5	1

Cerebellum

U_{CEB}

	NP-NC	LP-HC	J	p
TG	32	12	0·38	1
PG	8	0	0	1
PC	7	2	0·29	1
PC-O	6	2	0·33	1
SM	6	2	0·33	1
PI	5	2	0·5	1
PE	4	3	0·4	0·41
Cer	4	2	0·5	1
PS	3	1	0	0·17
PE-O	1	1	1	1

Right brain

U_{RiB}

	NP-NC	LP-HC	J	p
TG	0	1	0	1
PC	0	1	0	1
PS	1	4	0	0·07
PI	2	3	0·67	1
SM	0	1	0	1

$B_{SER-HEA}$

	NP-NC	LP-HC	J	p
TG	23	20	0·72	0·44
PC	2	0	0	1
LPC	1	3	0·33	1
PC-O	1	0	0	1
PE	1	1	0	0·25
LPE	1	1	1	1
SM	3	2	0·67	1
Cer	1	1	1	1

Heart

U_{HEA}

	NP-NC	LP-HC	J	p
TG	5	8	0·63	1
PI	0	3	0	1
PC-O	0	1	0	1
PG	1	0	0	1
CE	0	1	0	1

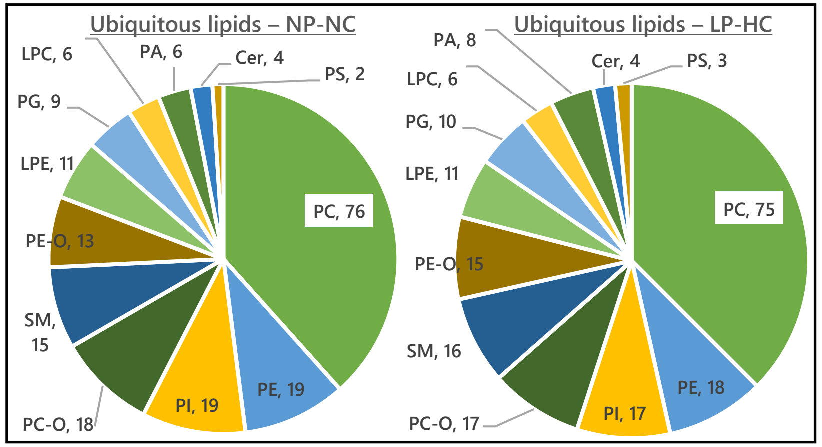

	J	p
PC	0·98	0·02
PE	0·95	1
PI	0·89	0·47
PC-O	0·94	1
SM	0·82	0·46
PE-O	0·87	1
LPE	1	1
PG	0·73	0·43
LPC	1	1
PA	0·75	1
Cer	1	1
PS	0·67	1

F1N, -ve

Liver

U_{LIV}

	NP-NC	LP-HC	J	p
Cer	3	5	0·6	1
PC	0	1	0	1
PG	0	1	0	1
PI	0	1	0	1
PS	1	0	0	1

$B_{LIV\text{-}SER}$

	NP-NC	LP-HC	J	p
PC	9	13	0·47	0·22
LPC	12	10	0·83	1
PC-O	4	4	1	1
SM	4	3	0·75	1
PI	3	3	0·5	0·43
PG	3	3	0·5	0·43
Cer	3	2	0·67	1
PE	2	1	0·5	1
LPE	1	1	1	1
PA	1	2	0·5	1
PE-O	0	1	0	1
PS	0	1	0	1

Serum

U_{SER}

	NP-NC	LP-HC	J	p
PC	2	3	0·33	0·43
LPC	2	4	0·50	1
PC-O	4	4	0·33	0·24
PE	2	0	0	1
PG	0	1	0	1
PI	1	0	0	1
PS	7	5	0·71	1
SM	1	0	0	1

$B_{SER\text{-}BRA}$

	NP-NC	LP-HC	J	p
PC	10	13	0·77	1
PC-O	9	11	0·82	1
PE	7	10	0·55	0·42
SM	7	6	0·63	0·48
PI	5	7	0·33	0·17
PG	2	5	0·25	0·42
Cer	1	2	0·50	1
PA	1	2	0·50	1
LPI	1	1	1	1
PE-O	0	6	0	1
PS	0	1	0	1

$B_{SER\text{-}HEA}$

	NP-NC	LP-HC	J	p
PC	13	9	0·47	0·22
LPC	15	13	0·75	0·47
PI	4	1	0	0·07
PC-O	5	3	0·33	0·32
PE	4	2	0·22	0·20
SM	4	3	0·17	0·12
LPE	1	1	1	1
PE-O	0	3	0	1
PG	4	4	0·60	0·43
PA	0	2	0	1
PS	0	2	0	1

Whole Brian

U_{BRA}

	NP-NC	LP-HC	J	p
PC	14	23	0·61	1
PC-O	8	13	0·62	1
CL	40	43	0·93	1
PA	1	1	1	1
Cer	2	2	1	1
PE	9	7	0·60	0·44
PE-O	14	15	0·71	0·45
PG	1	1	0	0·25
PI	4	3	0·75	1
PS	1	2	0	0·15
SM	0	2	0	1

Heart

U_{HEA}

	NP-NC	LP-HC	J	p
PI	10	8	0·64	0·46
PC	6	5	0·38	0·22
PC-O	2	0	0	1
PE	2	0	0	1
PG	3	2	0·67	1
PS	1	0	0	1
SM	1	1	1	1
Cer	1	0	0	1
PA	0	1	0	1

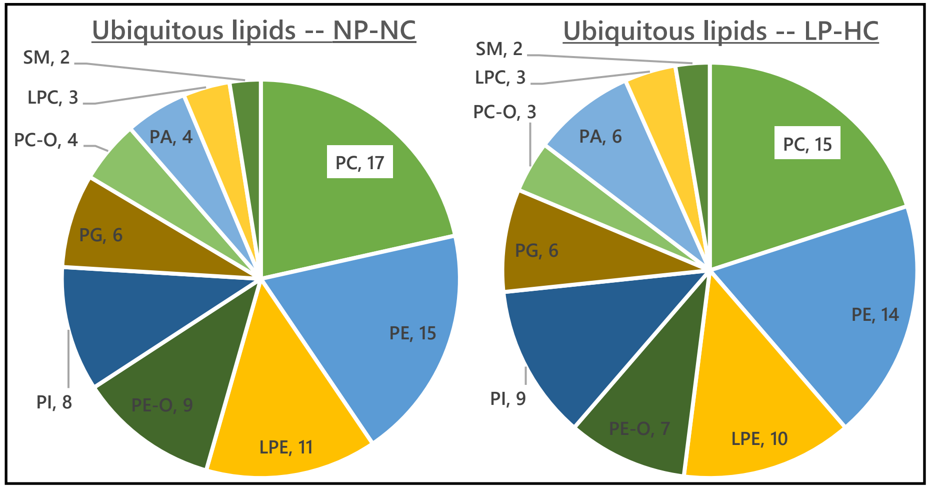

	J	p
PC	0·78	0·46
PE	0·93	1
LPE	0·91	1
PE-O	0·78	1
PI	0·7	1
PG	1	1
PC-O	0·75	1
PA	0·83	1
LPC	1	1
SM	1	1

F1A, -ve

Liver

Serum

Cerebellum

Right brain

Heart

Adipose

$B_{LIV-SER}$

	NP-NC	LP-HC	J	p
PC	55	55	0·84	0·49
PI	14	15	0·93	1
LPC	13	14	0·58	0·29
PC-O	12	15	0·69	0·47
SM	12	13	0·92	1
PE	8	8	0·78	0·43
PE-O	8	8	1	1
PG	4	5	0·80	1
PS	3	3	0·50	0·43
LPE	2	2	1	1
Cer	1	2	0·50	1
PA	1	3	0·33	1

U_{LIV}

	NP-NC	LP-HC	J	p
PC	2	4	0·20	0·22
Cer	3	2	0·67	1
PA	1	0	0	1
PC-O	0	1	0	1
PI	1	1	1	0·33
SM	1	1	1	1

U_{SER}

	NP-NC	LP-HC	J	p
PC	3	2	0·67	1
LPC	4	4	1	1
PC-O	2	4	0·2	0·22
PI	1	0	0	1
PS	7	7	1	1
SM	0	1	0	1

$B_{SER-CEB}$

	NP-NC	LP-HC	J	p
PC	48	50	0·89	0·47
PC-O	19	22	0·83	0·48
PE-O	14	17	0·82	1
PI	13	14	0·70	0·47
SM	13	15	0·87	1
PE	8	10	0·64	0·46
LPC	3	3	0·50	0·43
PG	3	3	0·75	1
LPE	1	1	0	1
PA	1	3	0·33	1
PS	1	2	0·50	1
Cer	0	1	0	1

$B_{SER-RiB}$

	NP-NC	LP-HC	J	p
PC	53	50	0·54	0·36
PC-O	20	20	0·67	0·48
PI	15	13	0·75	0·47
SM	14	15	0·81	0·47
PE	9	9	0·64	0·40
PE-O	8	11	0·72	1
LPC	5	1	0·20	1
PS	3	1	0·33	1
PG	5	2	0·40	1
LPE	1	0	0	1
LPI	1	0	0	1
PA	1	3	0·33	1
Cer	0	1	0	1

U_{Ceb}

	NP-NC	LP-HC	J	p
PC	2	4	0·20	0·22
PC-O	3	2	0·25	0·43
PE	1	1	0	1
PE-O	1	1	0	1
PI	0	1	1	1
SM	1	1	0	1
Cer	0	1	1	1

U_{RiB}

	NP-NC	LP-HC	J	p
PC	4	2	0·20	0·22
PE	1	1	1	1
PI	1	0	0	1

$B_{SER-HEA}$

	NP-NC	LP-HC	J	p
PC	56	51	0·83	0·48
PC-O	17	14	0·72	0·47
LPC	15	15	1	1
PI	13	14	0·87	1
SM	13	13	1	1
PE	8	5	0·63	1
PE-O	7	8	0·88	1
PG	4	4	1	1
LPE	2	2	1	1
PS	2	2	0·86	0·42
Cer	1	2	0·50	1
PA	1	3	0·33	1

U_{HEA}

	NP-NC	LP-HC	J	p
PC	6	3	0·29	0·25
PI	4	4	0·60	0·43
PI-O	3	1	0·33	1
PE	2	0	0	1
PE-O	1	1	1	1
PG	2	1	0·50	1
SM	1	1	1	1
PA	0	1	0	1
PC-O	0	1	0	1

$B_{SER-ADI}$

	NP-NC	LP-HC	J	p
LPC	5	3	0·60	0·32
PC	4	1	0	0·07
PE-O	3	1	0·33	1
LPE	2	1	0	1
PC-O	2	1	0·50	1
PE	2	1	0·50	1
PI	2	3	0·67	1
PS	2	1	0·50	1
SM	1	1	1	1
PA	0	2	0	1

U_{ADI}

	NP-NC	LP-HC	J	p
PI	8	6	0·75	1
PC-O	2	0	0	1
PC	1	1	1	1
LPC	1	1	1	1
PG	1	1	1	1
PS	1	0	0	1

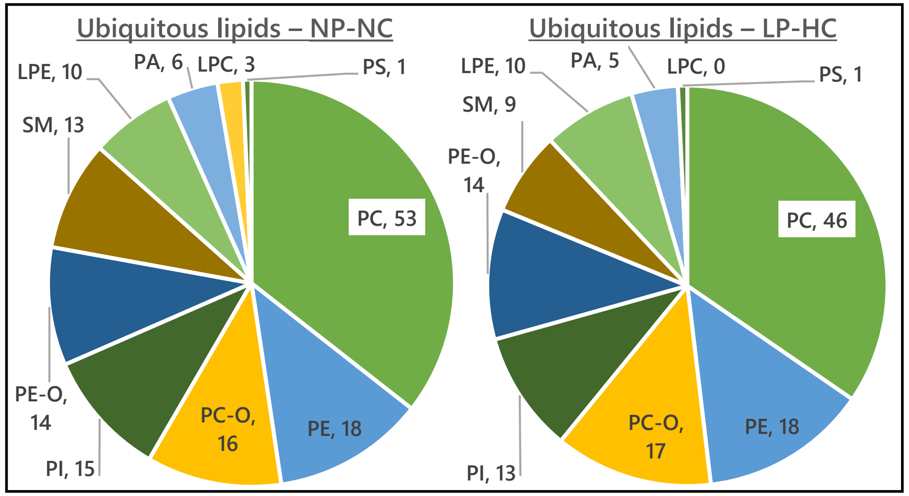

	J	p
PC	0·87	0·04
PE	1	1
PC-O	0·94	0·03
PI	0·87	1
PE-O	1	1
SM	0·69	1
LPE	0·82	0·42
PA	0·83	0·09
PG	1	1
LPC	0	1
PS	1	1

F2N -ve

Liver

U_{LIV}

	NP-NC	LP-HC	J	p
Cer	6	5	0·83	1
PC	4	5	0·13	0·09
PA	2	2	1	1
PG	0	1	0	1
PS	2	1	0·50	1
PI	2	1	0	0·14

$B_{LIV-SER}$

	NP-NC	LP-HC	J	p
PC	23	16	0·70	1
LPC	19	18	0·85	0·47
PI	7	4	0·57	1
SM	5	4	0·80	1
LPE	3	3	0·75	0·16
PC-O	3	2	0·67	1
PE	3	3	1	1
PG	3	3	1	1
PA	2	3	0·67	1
PS	2	2	1	1
Cer	1	1	1	1
PE-O	1	0	0	1
LPI	0	1	0	1

Serum

U_{SER}

	NP-NC	LP-HC	J	p
PC	1	1	0	0·25
PC-O	3	3	0·50	0·43
PS	3	4	0·75	1
LPC	2	3	0·67	1
SM	1	1	1	1
PI	0	1	0	1

$B_{SER-CEB}$

	NP-NC	LP-HC	J	p
PC	13	8	0·31	0·08
PC-O	13	9	0·68	1
PI	7	5	0·71	1
LPC	6	8	0·75	1
SM	6	5	0·83	1
PE	4	4	1	1
PE-O	3	1	0·33	1
PS	2	2	1	1
LPE	1	2	0·5	1
LPI	1	1	1	1
PA	0	1	0	1
Cer	1	1	0	1

$B_{SER-RiB}$

	NP-NC	LP-HC	J	p
PC	20	15	0·67	0·47
LPC	11	7	0·64	1
PE	10	10	1	1
PC-O	9	6	0·67	1
PI	8	5	0·63	1
SM	5	6	0·57	0·44
PS	3	3	1	1
PE-O	3	1	0·33	1
LPE	2	2	1	1
PA	2	3	0·67	1
LPI	1	1	1	1
Cer	1	1	0	1

Cerebellum

U_{CEB}

	NP-NC	LP-HC	J	p
PC	8	6	0·75	1
PC-O	5	7	0·71	1
PE	3	3	1	1
PE-O	1	2	0·50	1
SM	1	2	0·50	1
PG	1	0	0	1
PI-O	1	1	1	1
Cer	0	1	0	1

Right brain

U_{RiB}

	NP-NC	LP-HC
-	-	-

$B_{SER-HEA}$

	NP-NC	LP-HC	J	p
PC	12	4	0·14	0·04
LPE	11	10	0·91	1
LPC	10	6	0·45	0·38
PI	4	0	0	0
SM	4	2	0	0·06
PA	2	1	0	0·15
PC-O	1	3	0·33	1
PG	1	1	0·25	0
PS	1	1	1	1
Cer	1	0	0	1

Heart

U_{HEA}

	NP-NC	LP-HC	J	p
PC	4	6	0·43	0·39
PC-O	1	2	0·50	1
PE	0	2	0	1
PI	8	8	1	1
PS	1	0	0	1
