# Supplementary material for: Lipid Traffic Analysis reveals the impact of high paternal carbohydrate intake on offsprings’ lipid metabolism
Source: Commun Biol. 2021 Feb 5;4:163. doi: 10.1038/s42003-021-01686-1 (PMC7864968; doi:10.1038/s42003-021-01686-1)

Lipid Traffic Analysis reveals the impact of high paternal carbohydrate intake on offsprings' lipid metabolism

Supplementary data 2

Furse ***et al.***

Sections

- Assignments
- Samples
- Degradation test

Assignments

Shift (ppm)	Assignment
0·00	Phosphatidylcholine*
0·05	plasmalogen-Phosphatidylcholine
0·18-0·22	*Unknown*
0·45	*lyso*-Phosphatidylcholine
0·52	Phosphatidylserine
0·55	Phosphatidylethanolamine**
0·58	plasmalogen-Phosphatidylethanolamine
0·78	Cardiolipin
0·83	Sphingomyelin
0·91	*lyso*-Phosphatidylethanolamine
1·08-1·12	Phosphatidylinositol
1·23	Phosphatidylglycerol
1·53	*lyso*-Phosphatidylinositol
1·72	*lyso*-Phosphatidylglycerol
4·80-5·50	Phosphatidic acid**
6·00-6·50	*lyso*-Phosphatidic acid

*PC is 0·00 ppm by definition, *i.e.* data were referenced to give the PC signal at 0·00 ppm.

** PE and PA have several resonances, due to interactions with the solvent system and several possible adducts

References
- Bosco *et al.* 1997, Anal. Biochem. DOI: 10.1006/abio.1996.9907
- Culeddu *et al.* 1998, Magn. Res. Chem. DOI: 10.1002/(sici)1097-458x(199812)36:12<907::aid-omr394>3.0.co;2-5
- Murgia, *et al.* 2003, Lipids, DOI: 10.1007/s11745-003-1500-3
- Cremonini *et al.* 2004, J. Sci. Food Agri. DOI: 10.1002/jsfa.1683
- Furse *et al.* 2013, *J. Chem Biol*, DOI: 10.1007/s12154-012-0090-1

Samples

- Liver
  - F1N
    - NP-NC
    - LP-HC
  - F1A
    - NP-NC
    - LP-HC
  - F2N
    - NP-NC
    - LP-HC

- Heart
  - F1N
    - NP-NC
    - LP-HC
  - F1A
    - NP-NC
    - LP-HC
  - F2N
    - NP-NC
    - LP-HC

- Adipose
  - F1A
    - NP-NC
    - LP-HC
    - LP-HC (petrol washed)

- Serum
  - F2N (pooled)

- Right Brain
  - F1A (pooled)

- Cerebellum
  - F1A male
  - F2N female

Liver
F1N
NP-NC

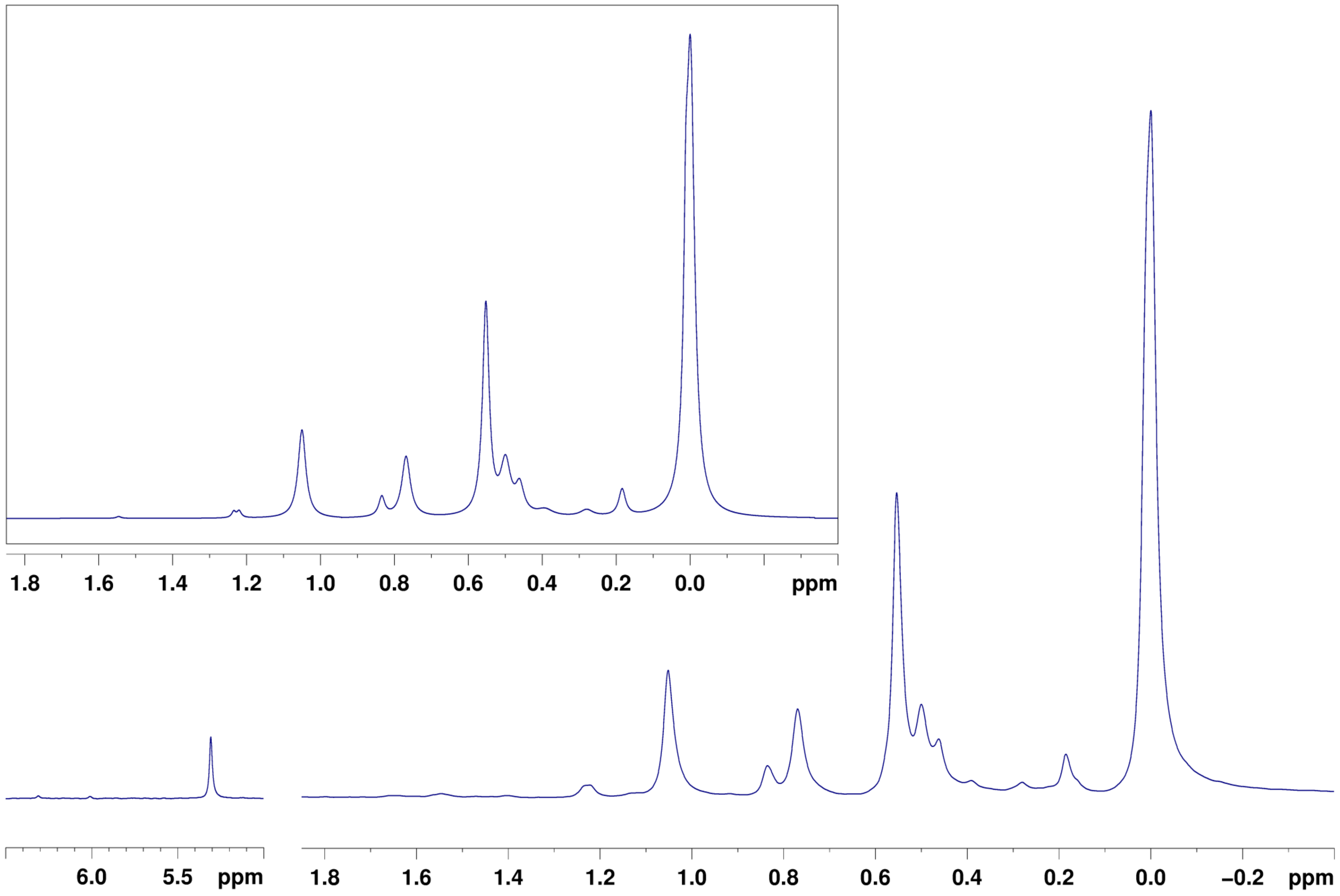

Liver
F1N
LP-HC

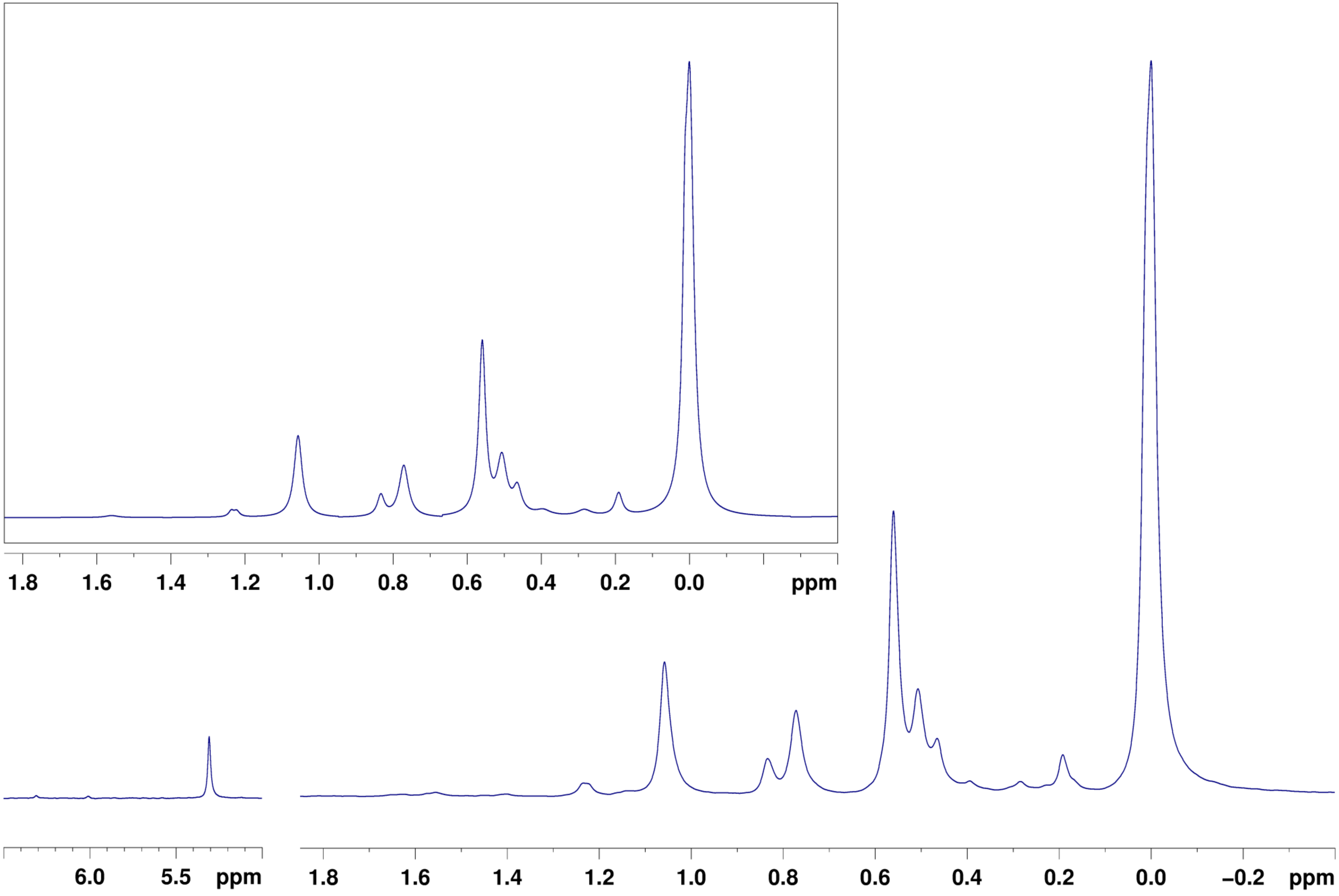

Liver
F1A
NP-NC

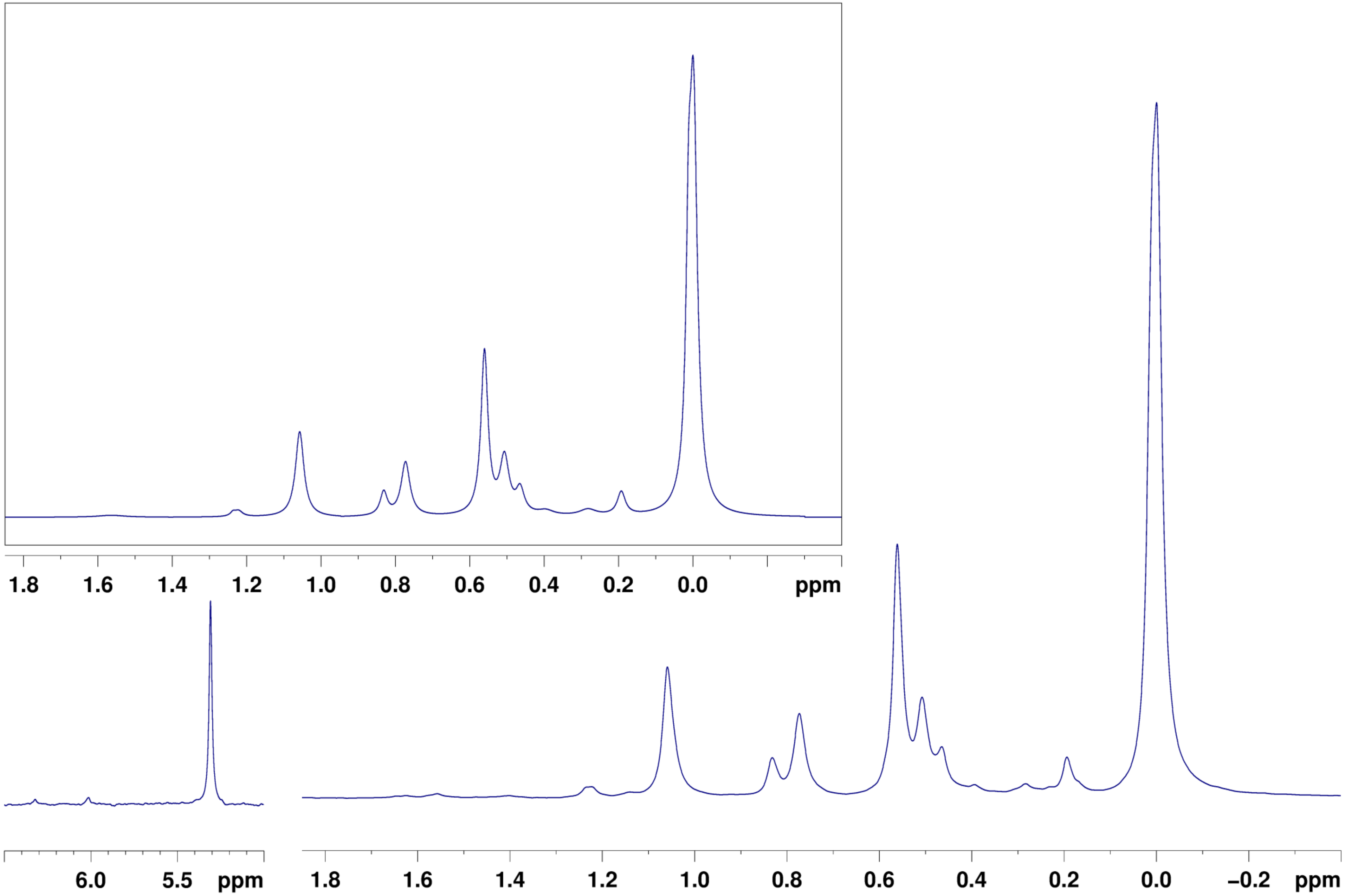

Liver
F1A
LP-HC

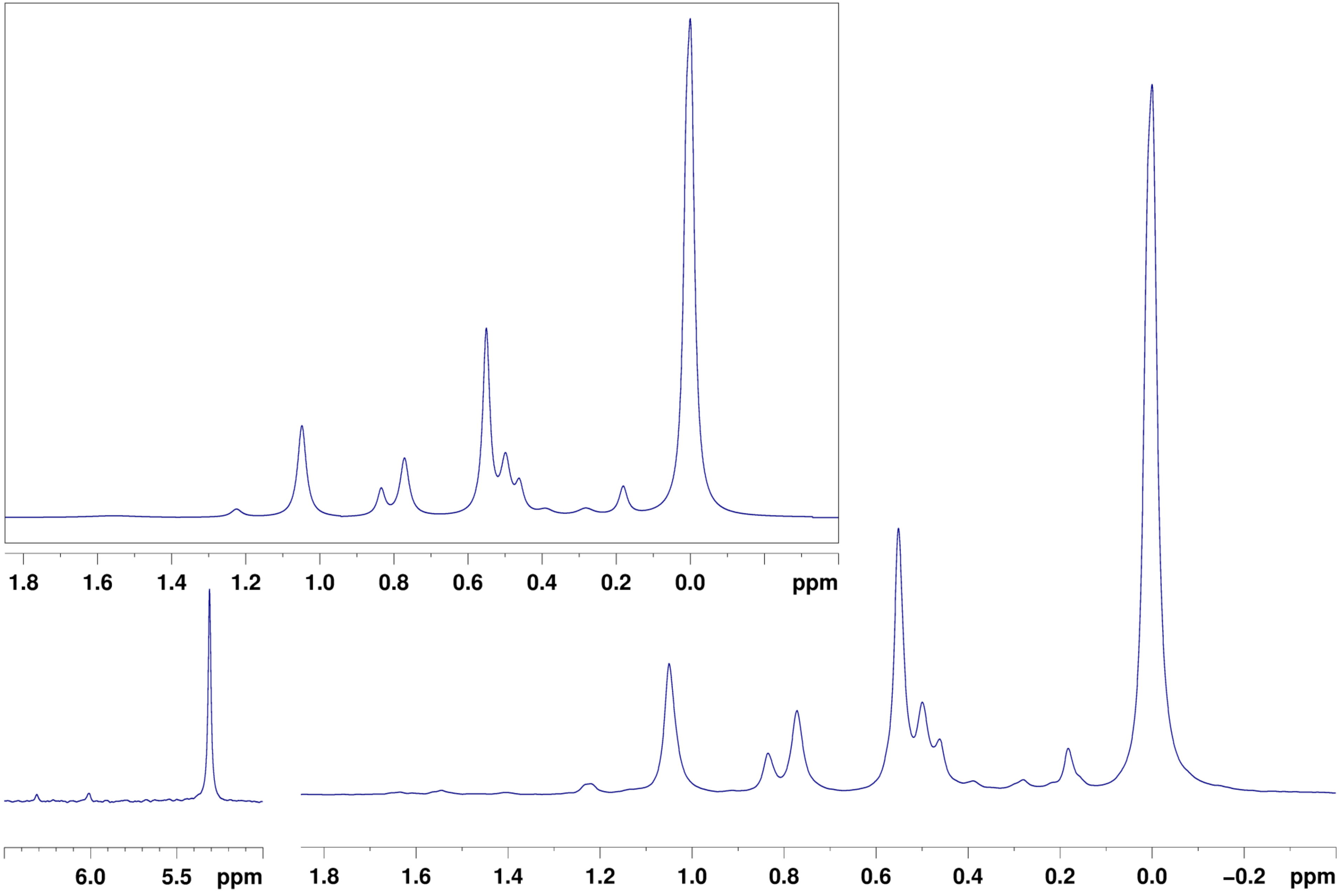

Liver
F2N
NP-NC

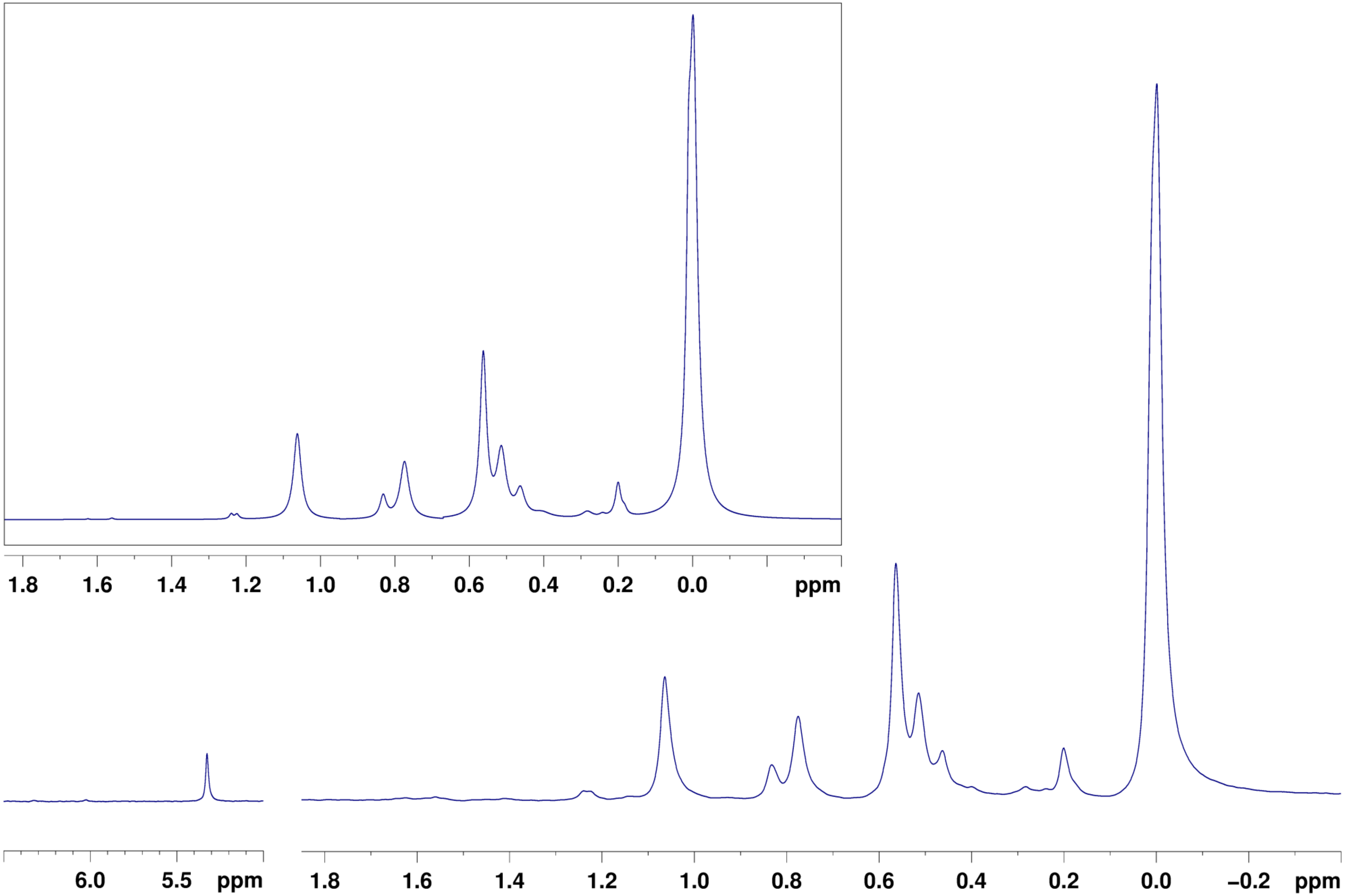

Liver
F2N
LP-HC

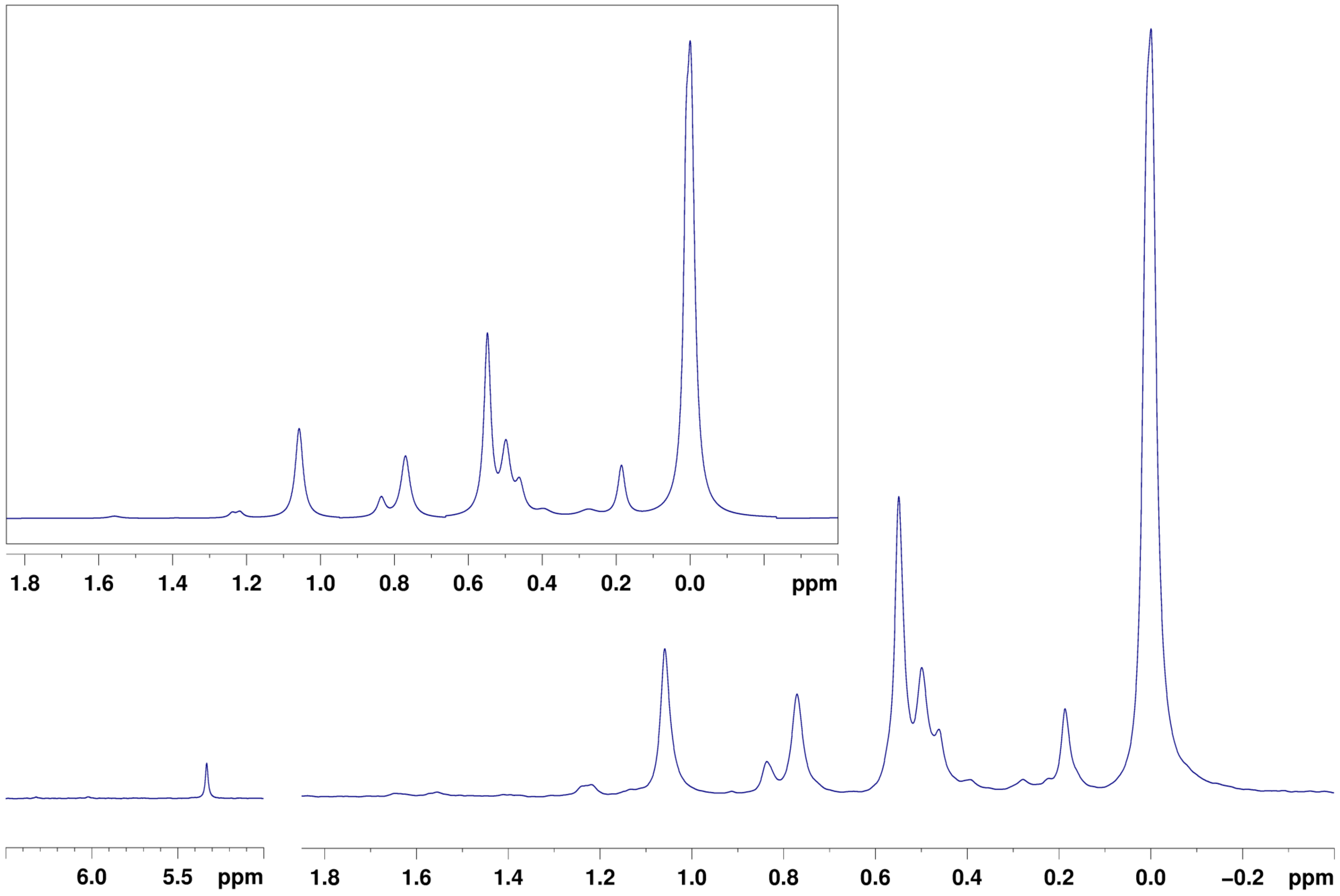

Heart F1N NP-NC

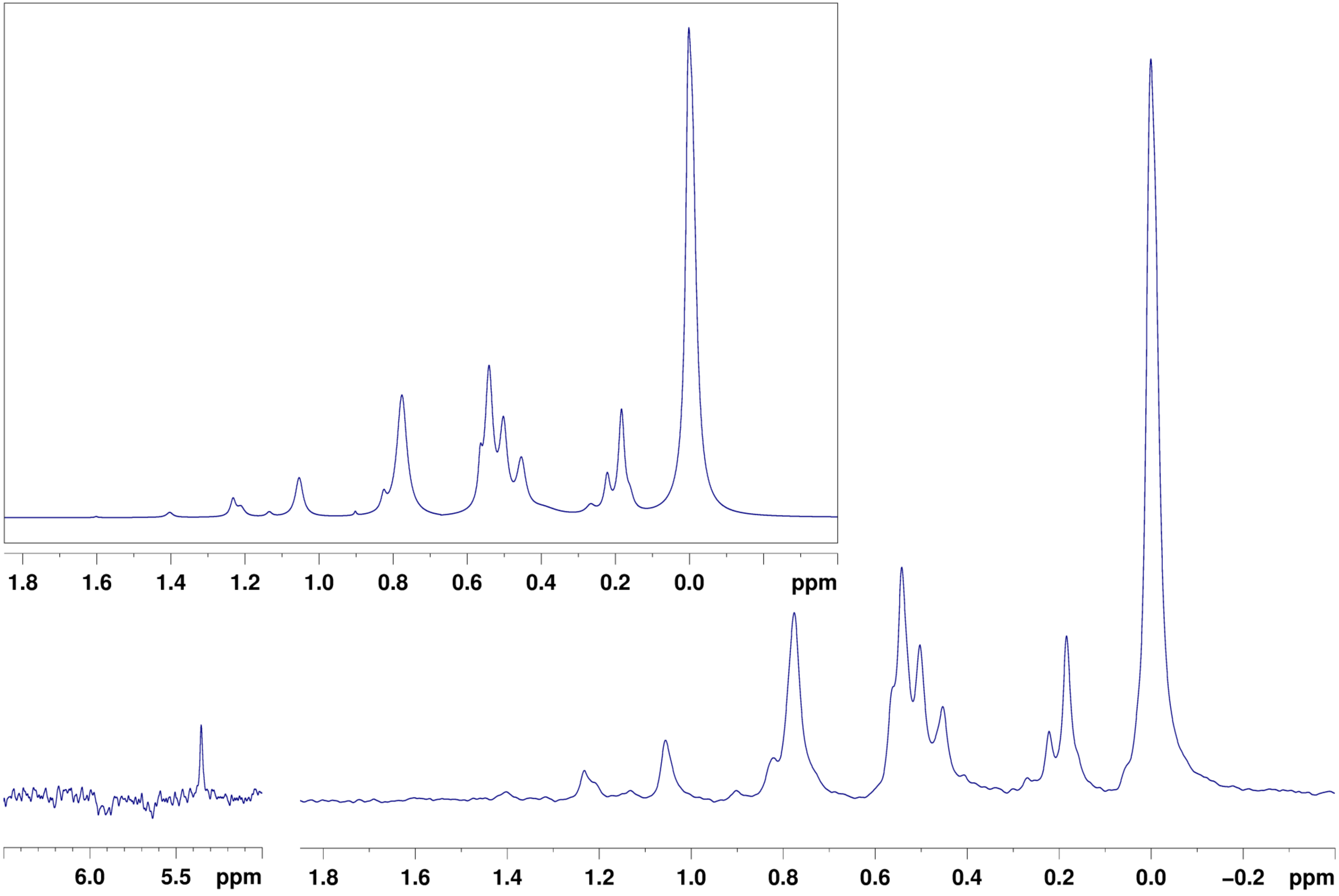

Heart
F1N
LP-HC

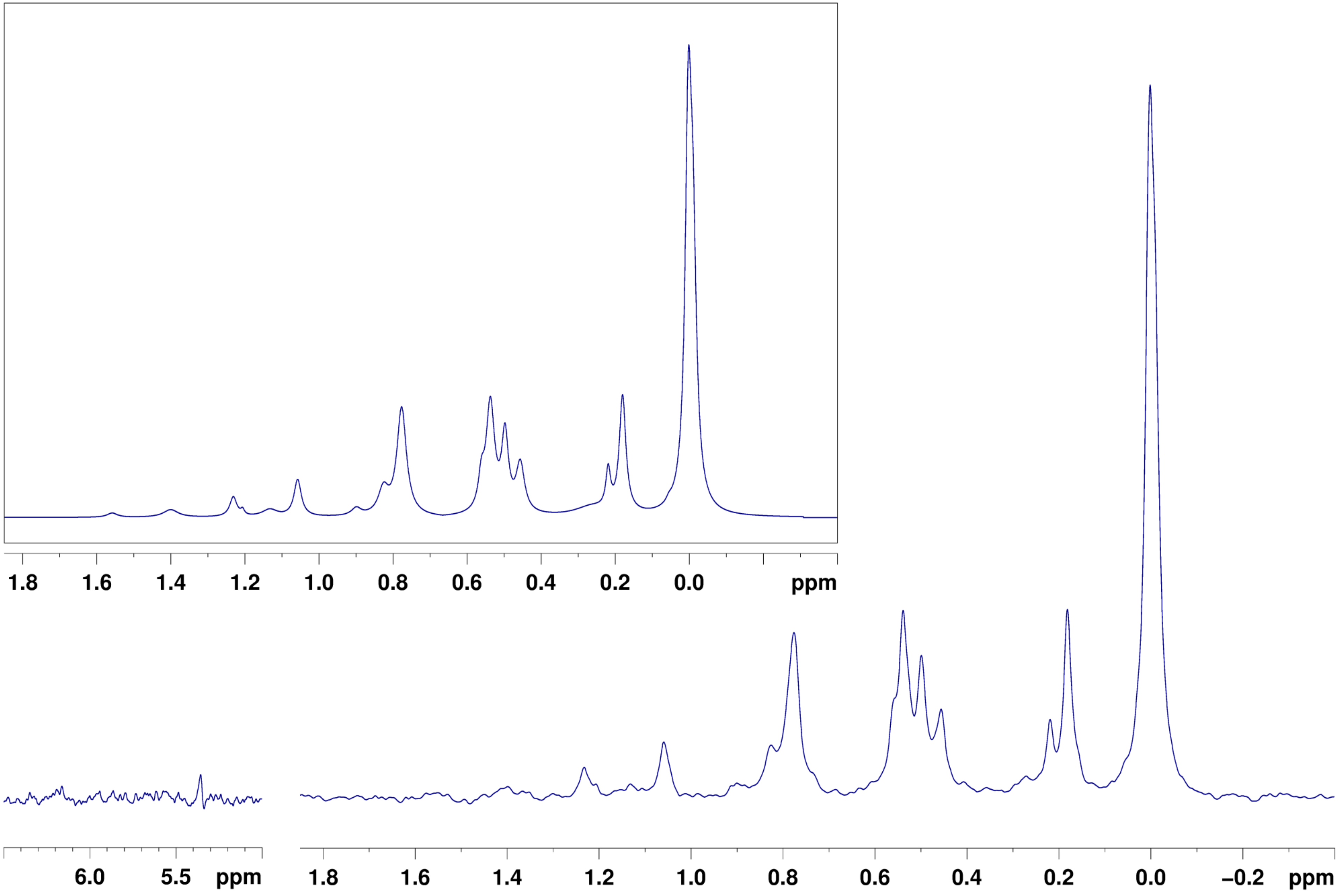

Heart
F1A
NP-NC

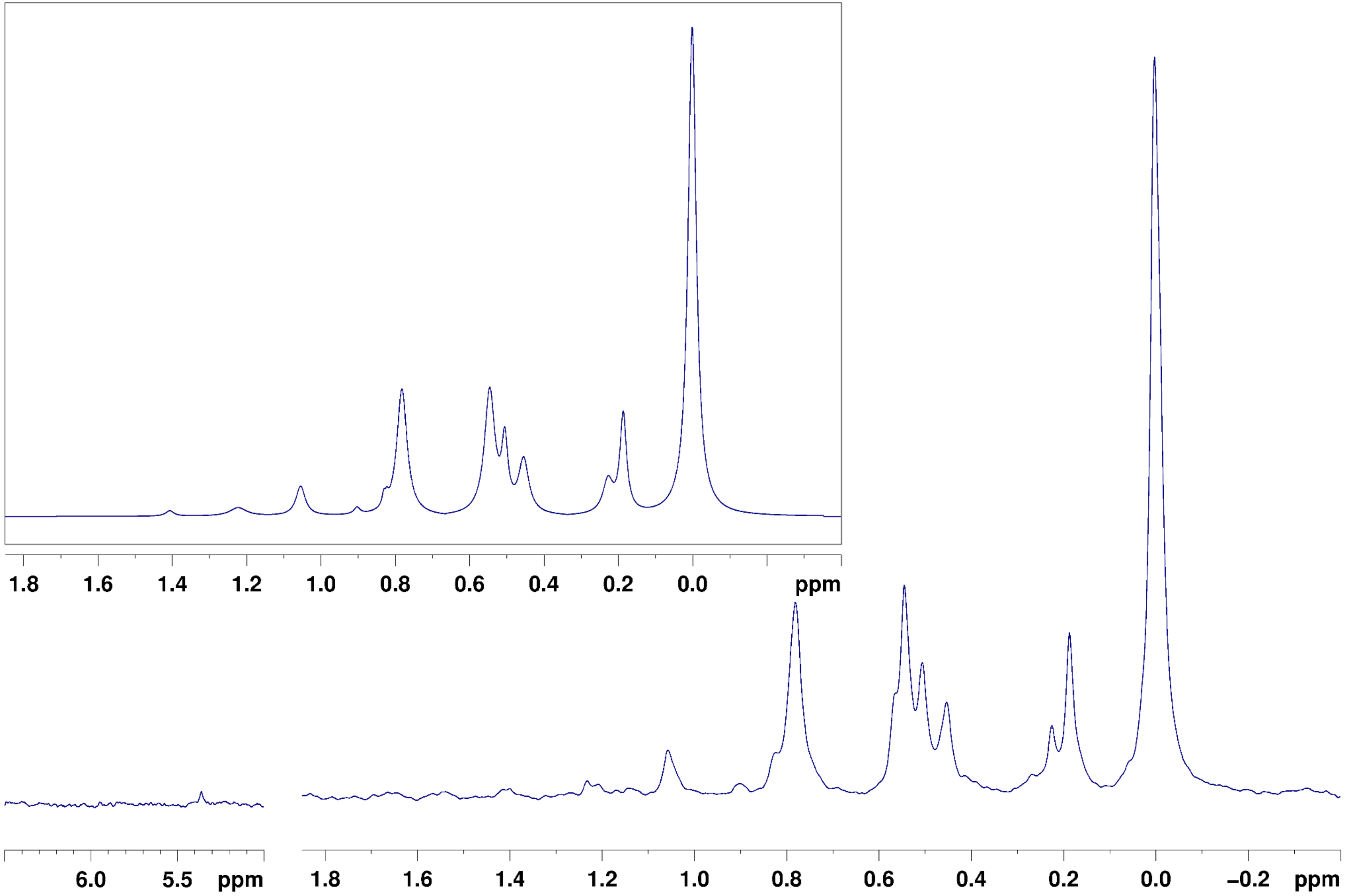

Heart
F1A
LP-HC

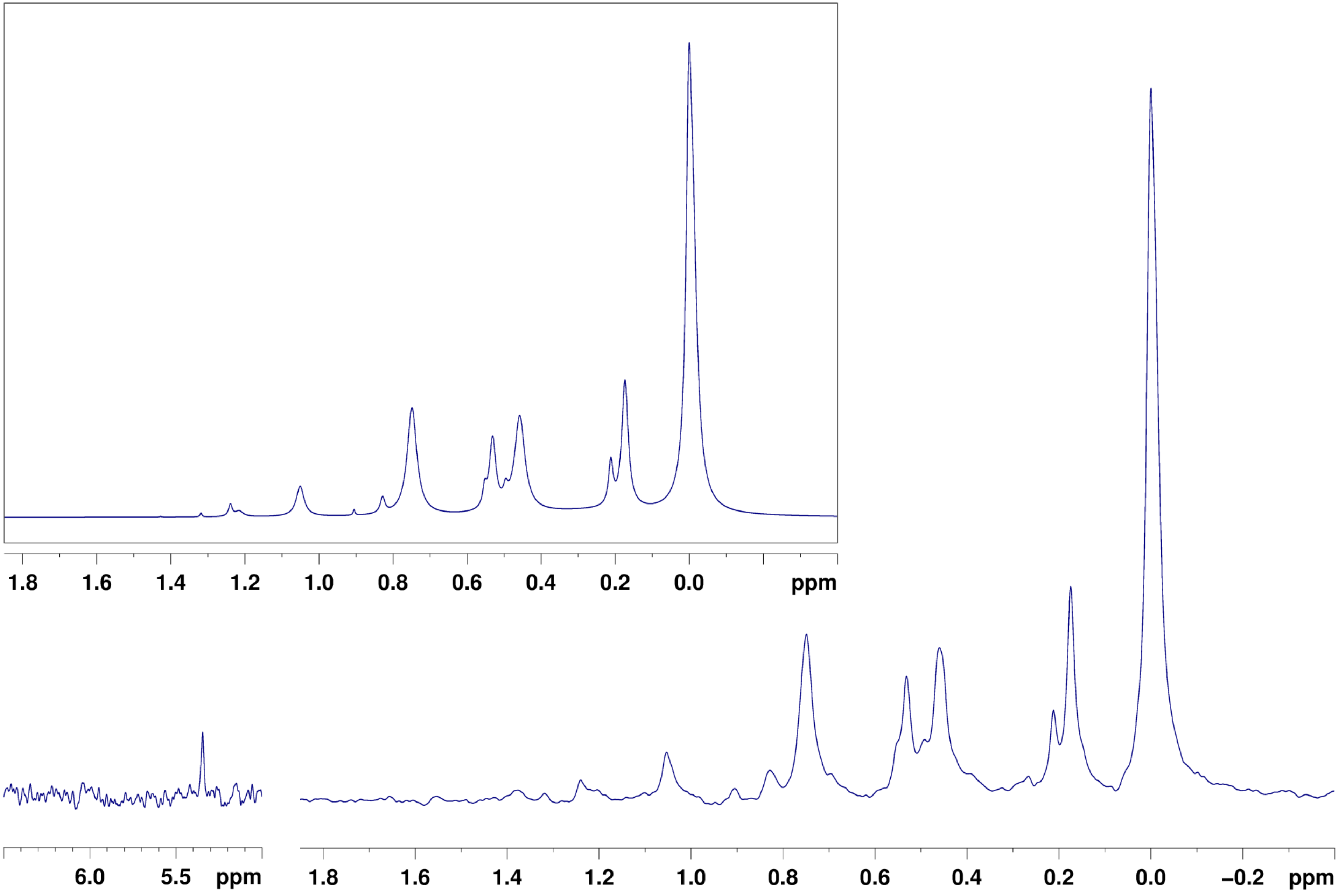

Heart
F2N
NP-NC

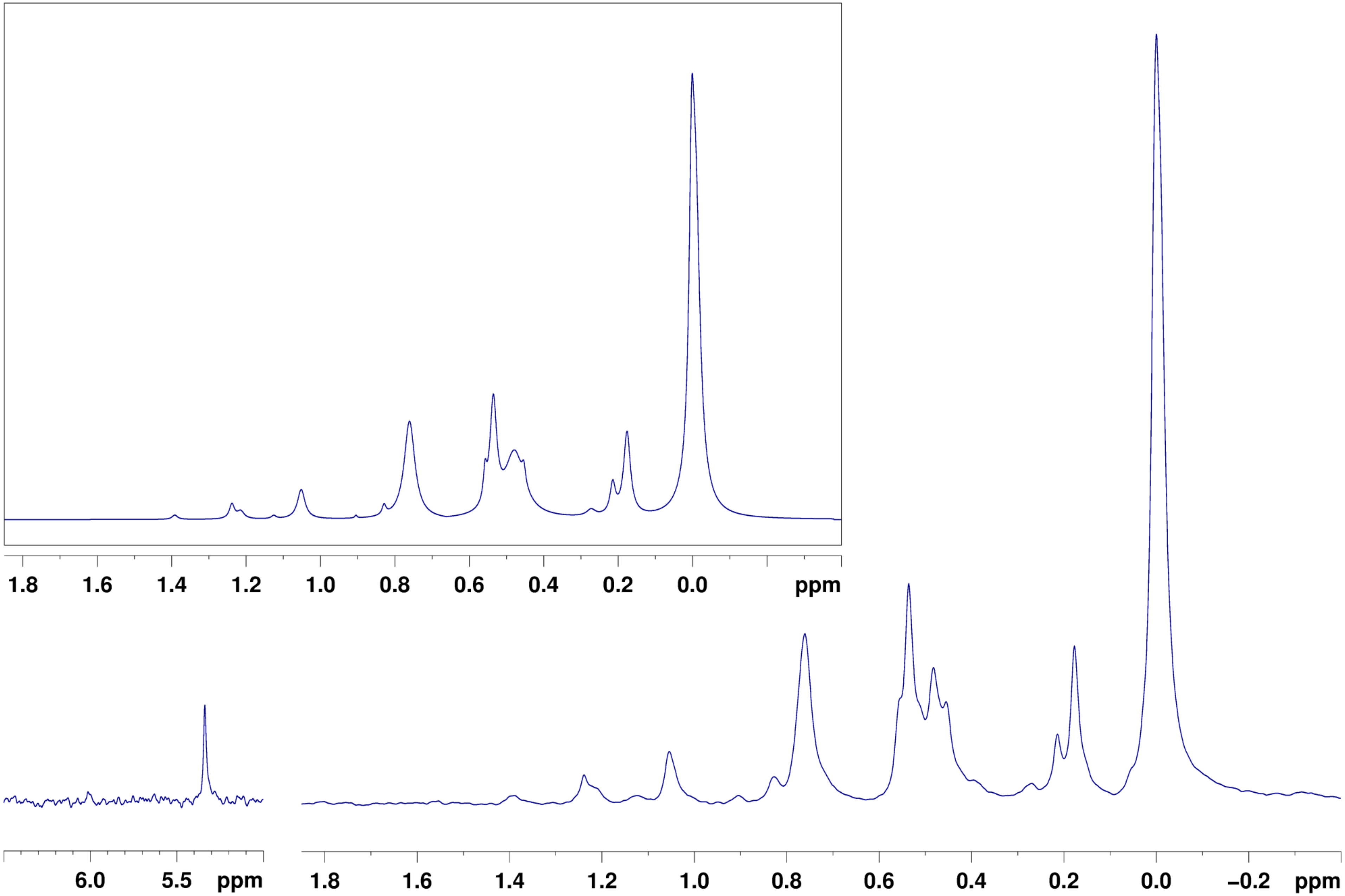

Heart
F2N
LP-HC

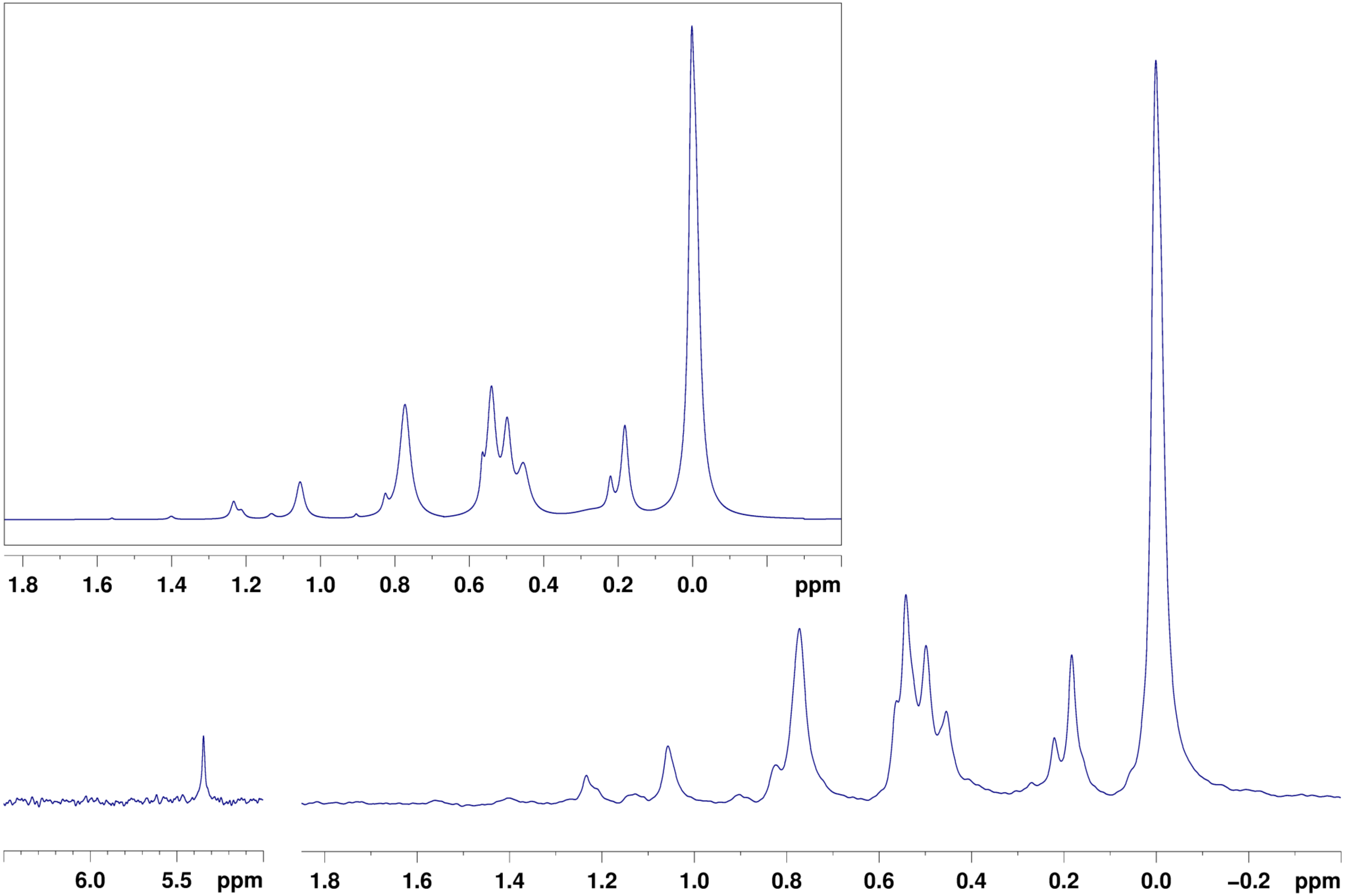

Adipose
F1A
NP-NC

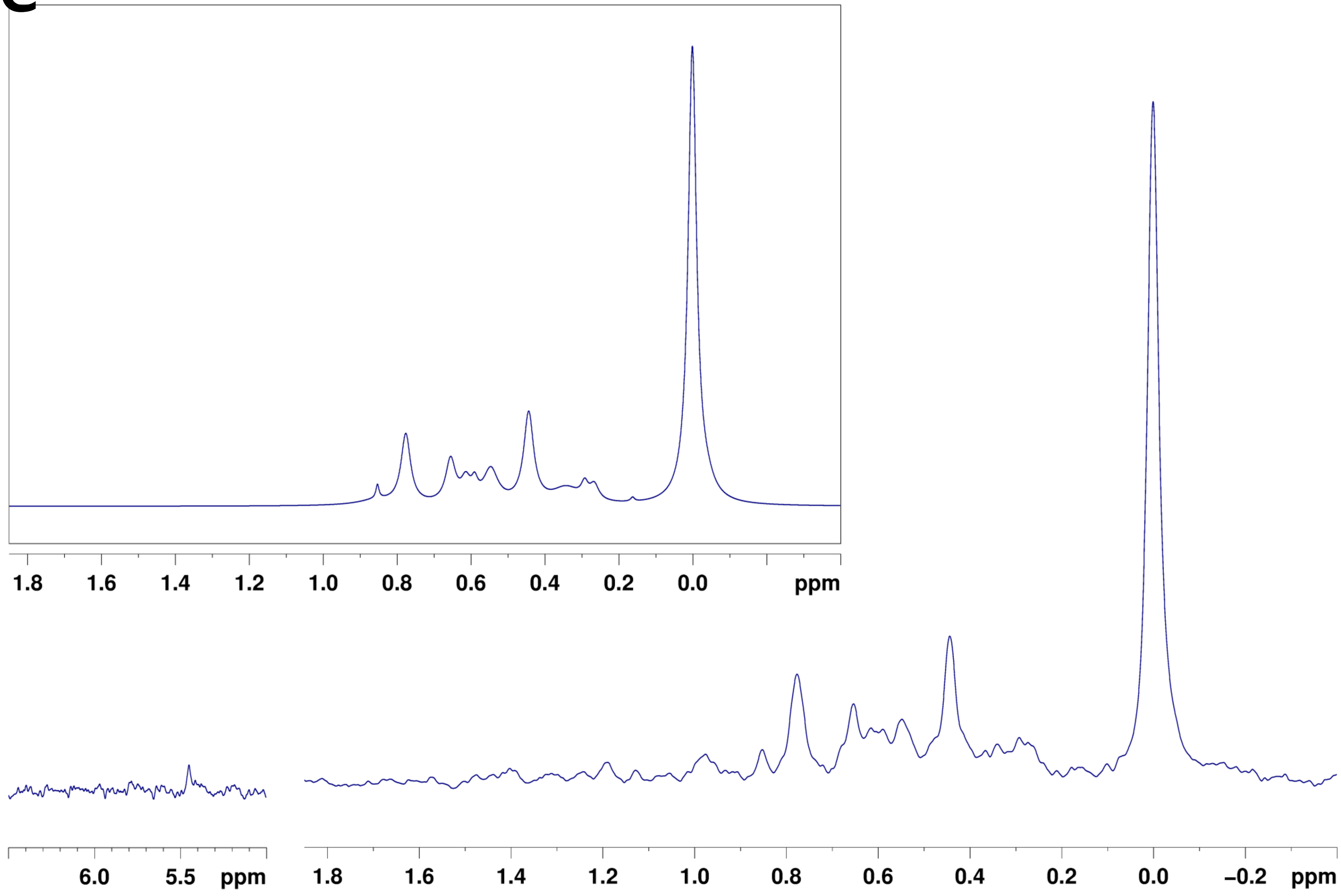

Adipose
F1A
LP-HC

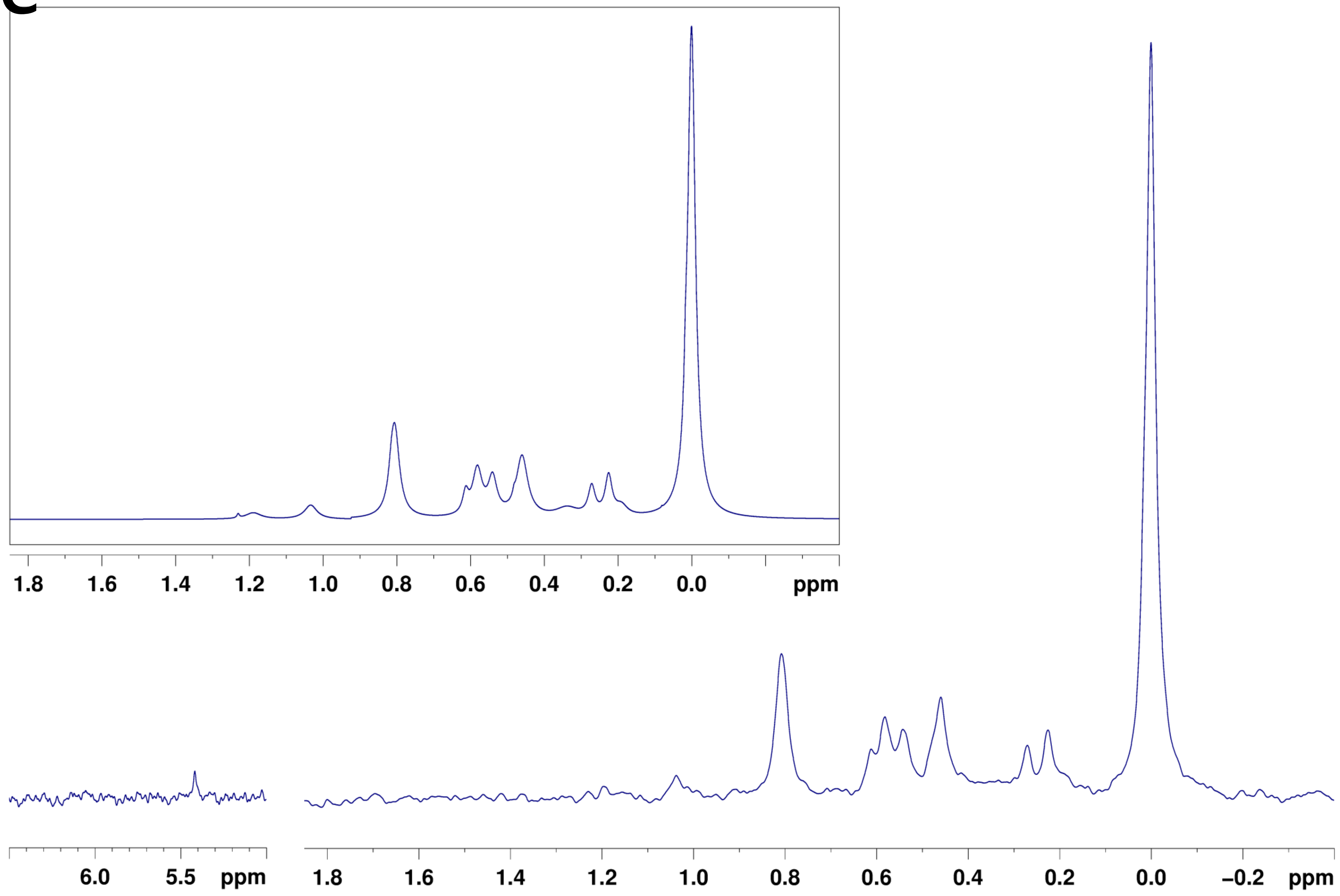

Adipose
F1A
LP-HC
PW

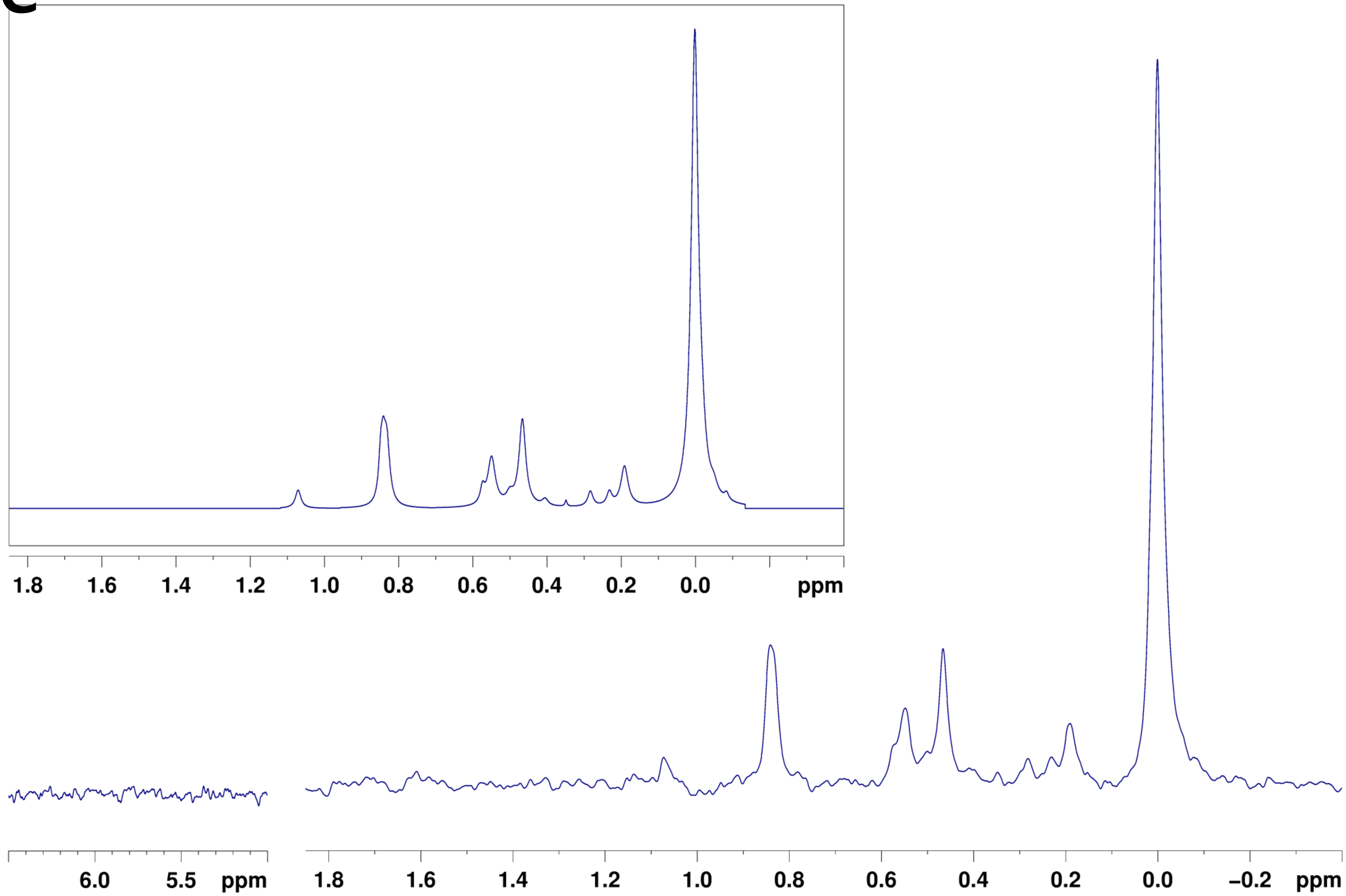

Serum
F2N
Male

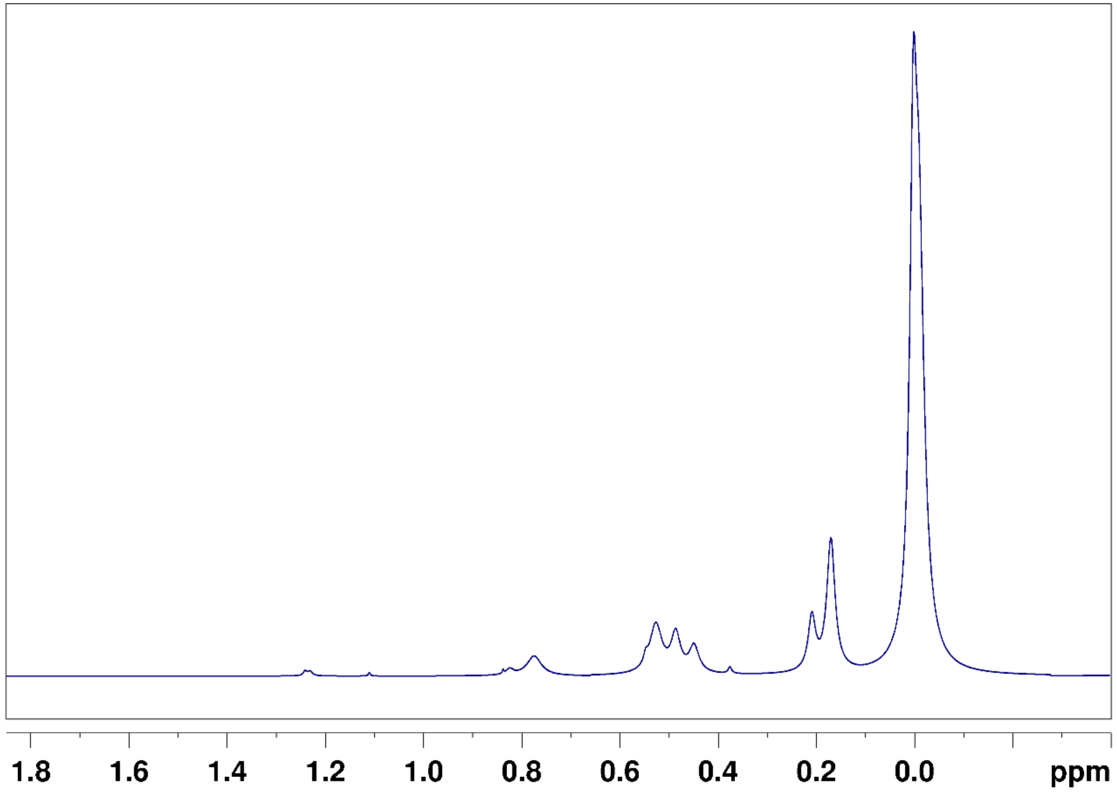

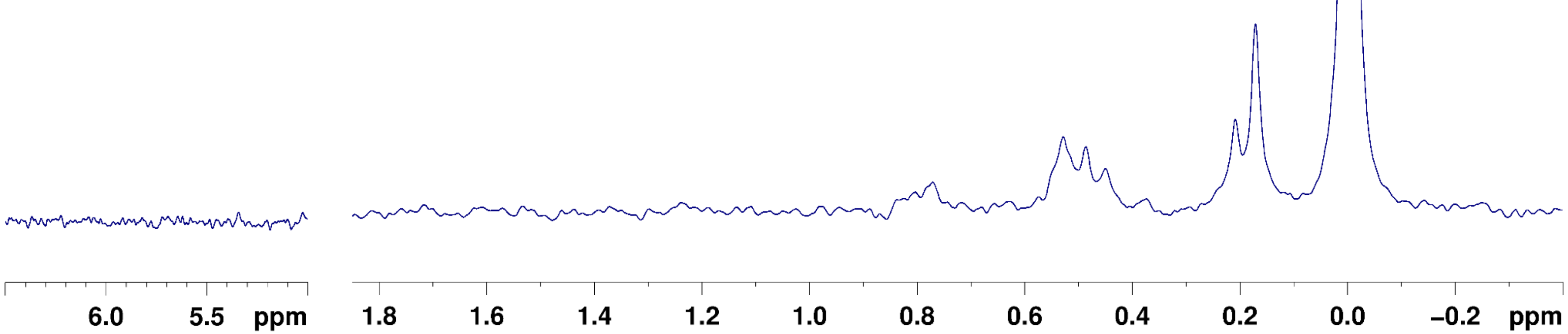

Right Brain
F1A
Pooled

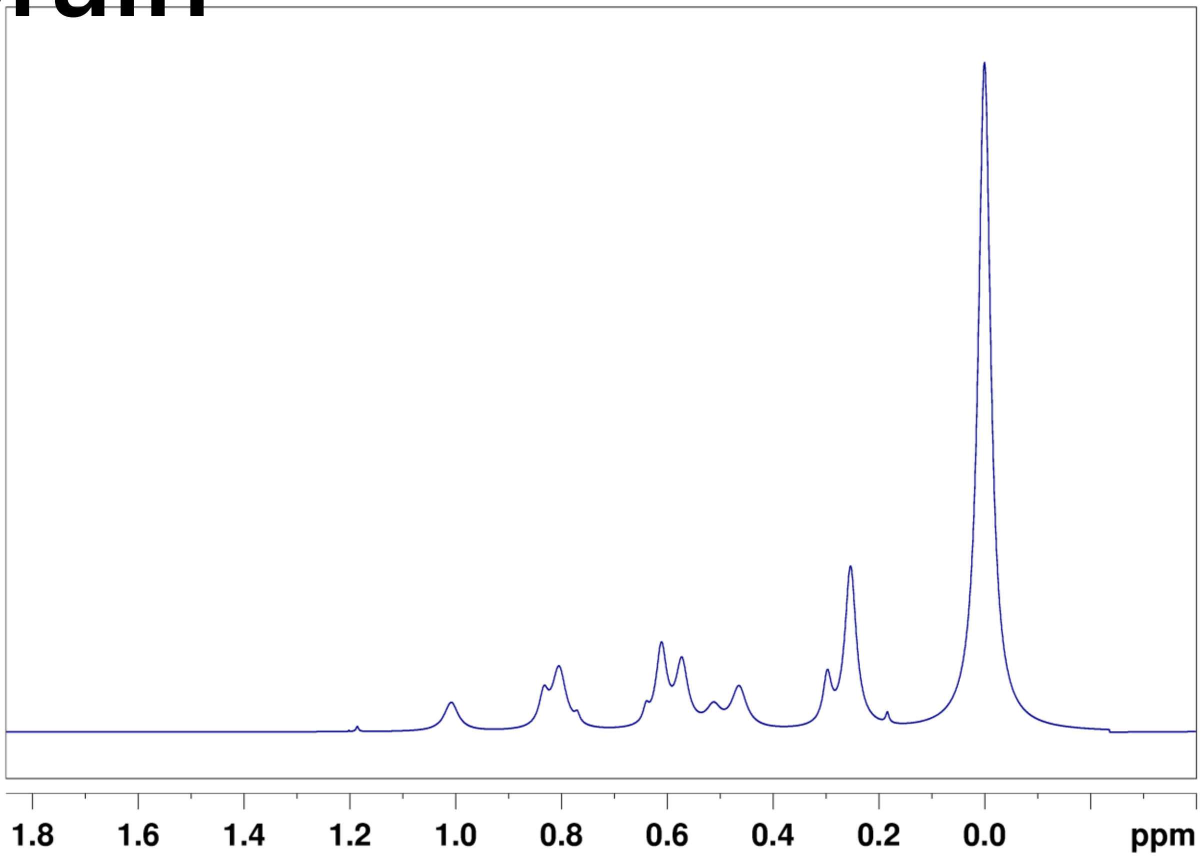

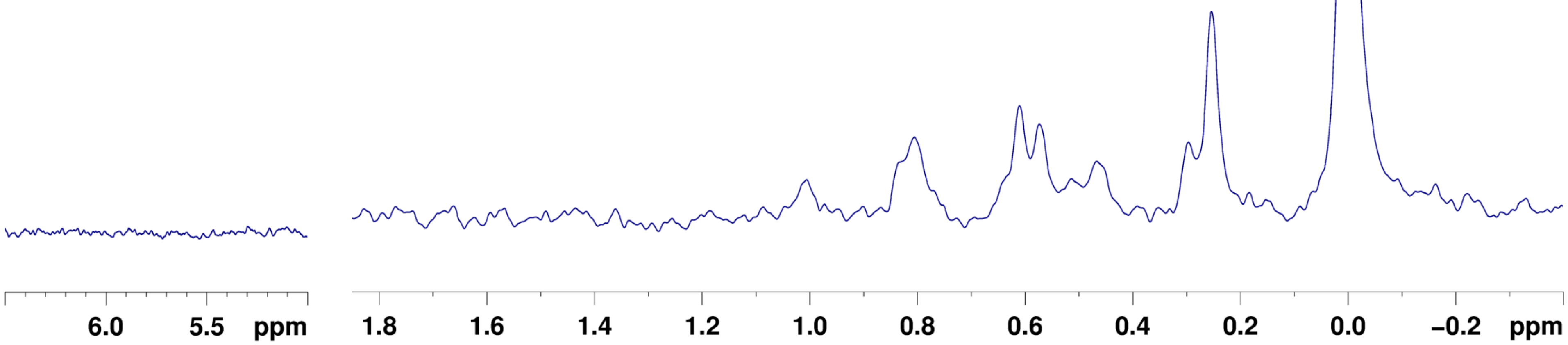

Cerebellum
F1A
Male

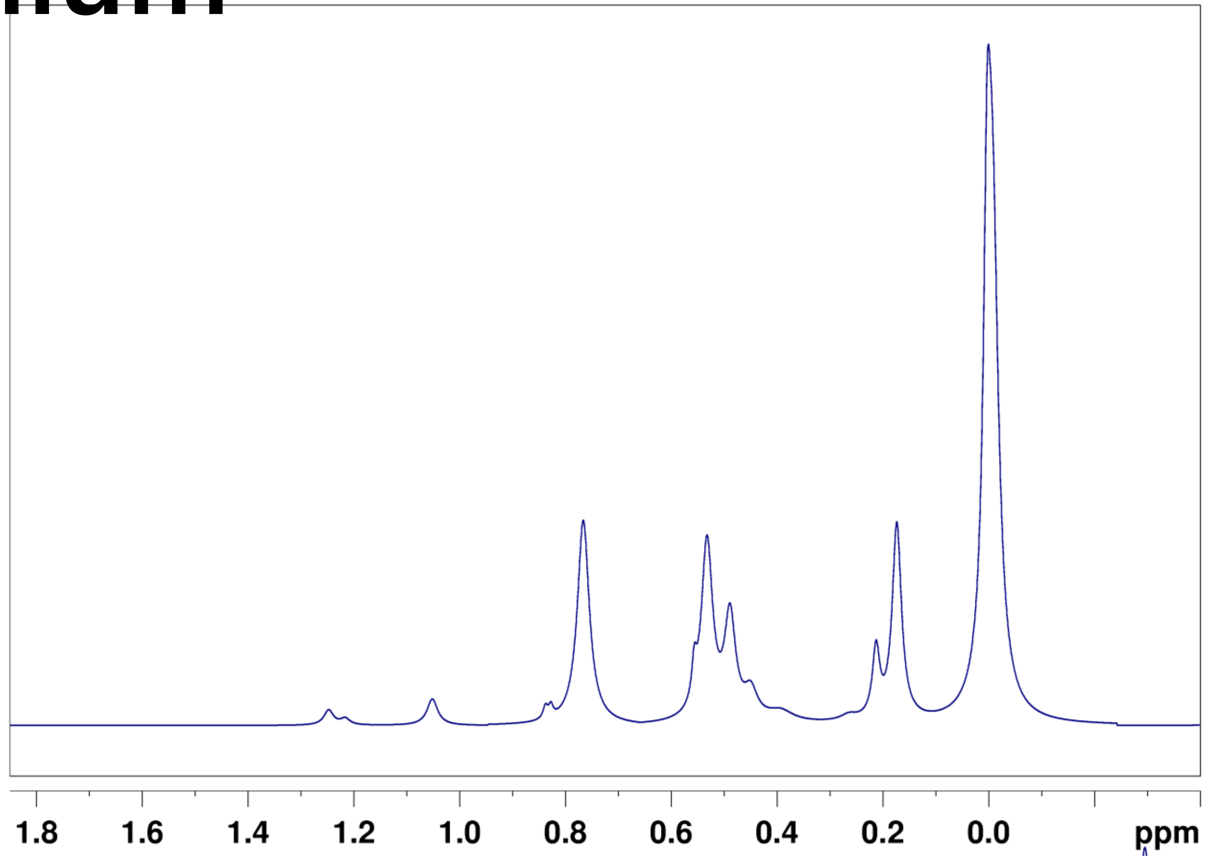

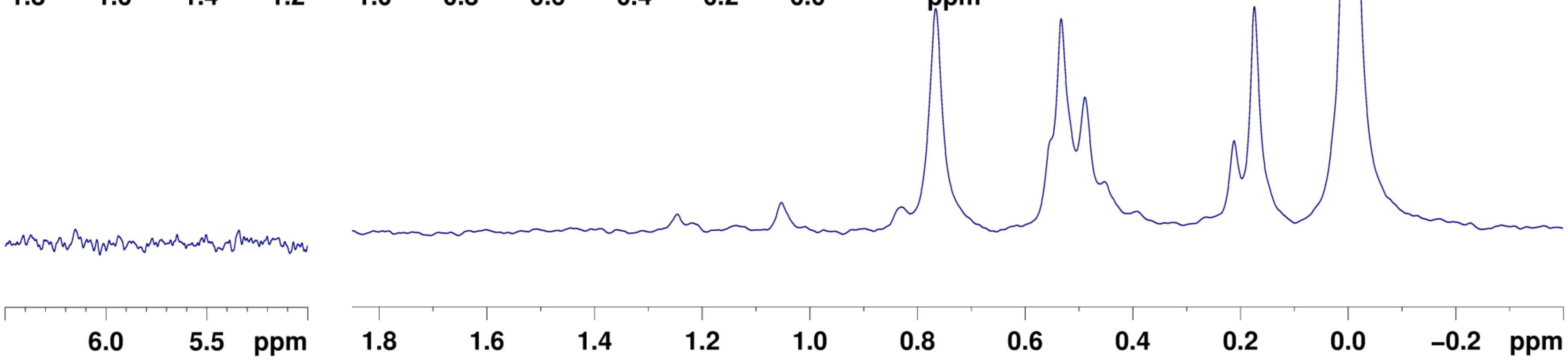

Cerebellum
F2N
Female

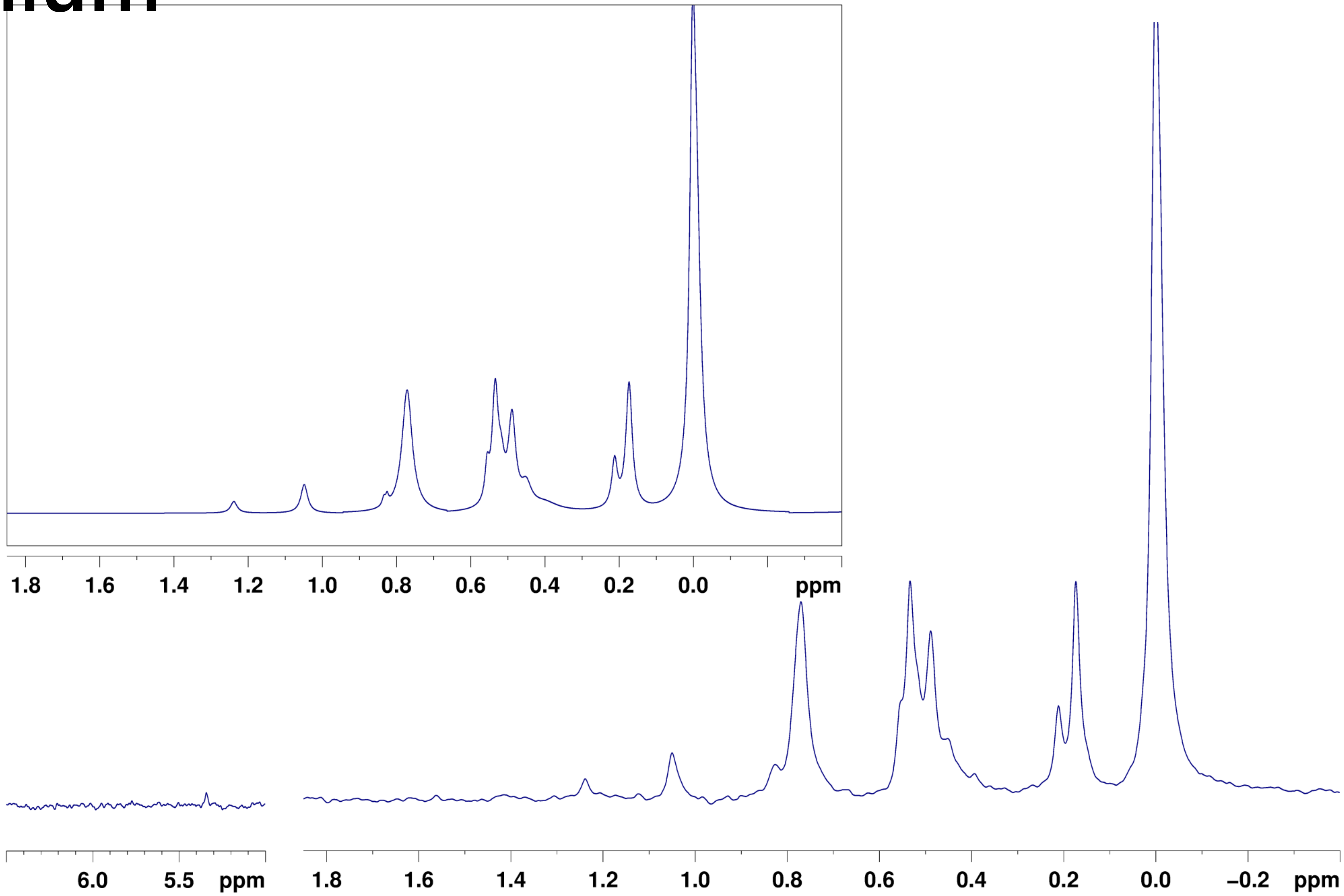

Degradation test

- F2N, Liver, NP-NC
    - 0h (red trace)
    - +48h (blue trace)

Liver
F2N
NP-NC
0h, +48h

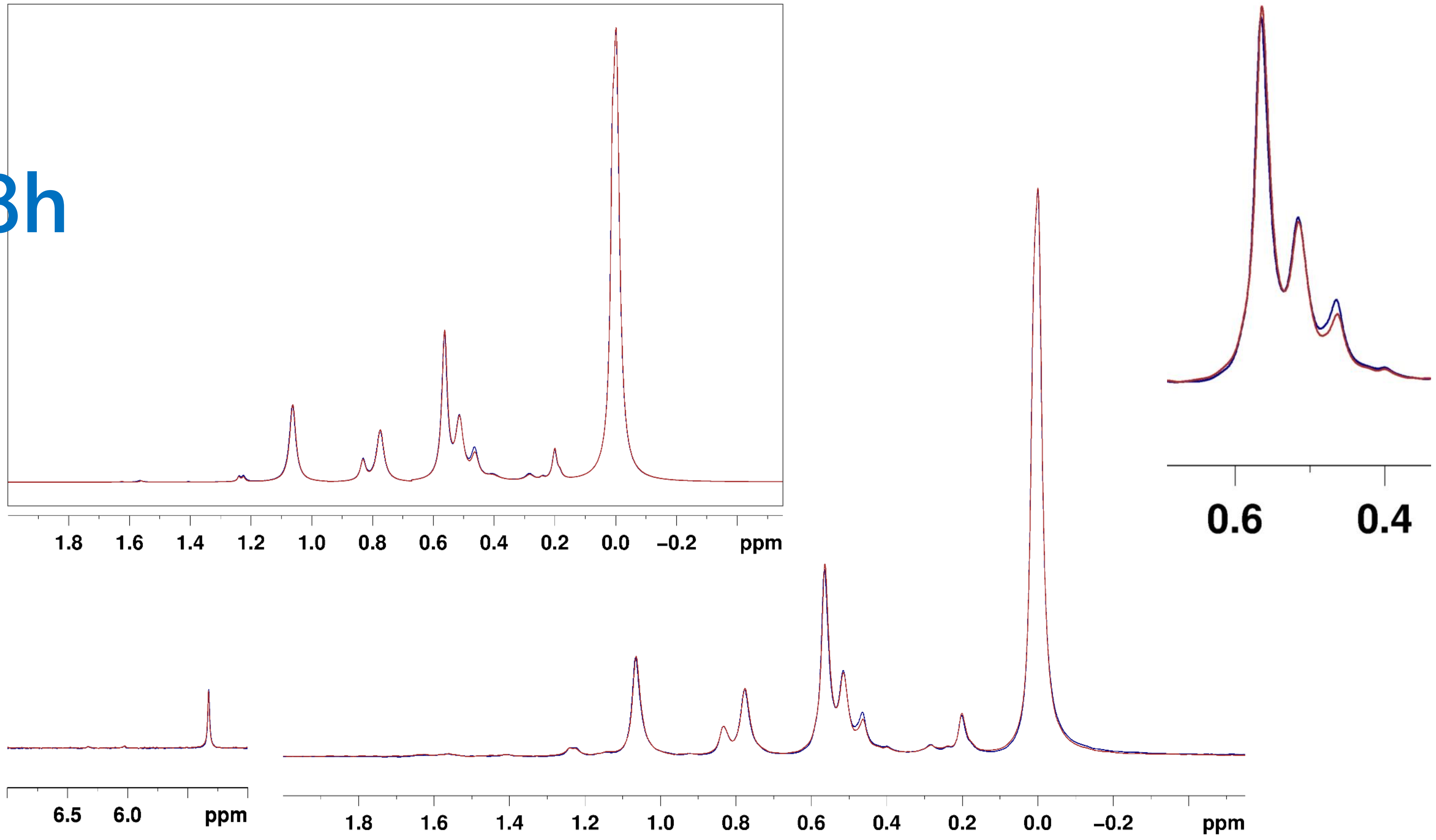

Supplement: Supplementary file 5 — Supplementary Data 2 [file 42003_2021_1686_MOESM5_ESM.pdf]
