# Supplementary material for: Lipid Traffic Analysis reveals the impact of high paternal carbohydrate intake on offsprings’ lipid metabolism
Source: Commun Biol. 2021 Feb 5;4:163. doi: 10.1038/s42003-021-01686-1 (PMC7864968; doi:10.1038/s42003-021-01686-1)
Supplement: Supplementary file 8 — Supplementary Data 5 [file 42003_2021_1686_MOESM8_ESM.pdf]

Negative ionisation mode

m/z	Lipid variable	Isobar 1	Isobar 2
568.4946	Cer(32:1)_[M+OAC]1-		
596.5259	Cer(34:1)_[M+OAC]1-		
536.5048	Cer(34:1)_[M-H]1-		
564.5361	Cer(36:1)_[M-H]1-		
562.5205	Cer(36:2)_[M-H]1-		
592.5674	Cer(38:1)_[M-H]1-		
590.5518	Cer(38:2)_[M-H]1-		
666.6042	Cer(39:1)_[M+OAC]1-		
680.6198	Cer(40:1)_[M+OAC]1-		
620.5987	Cer(40:1)_[M-H]1-		
618.5831	Cer(40:2)_[M-H]1-		
694.6355	Cer(41:1)_[M+OAC]1-		
634.6144	Cer(41:1)_[M-H]1-		
692.6198	Cer(41:2)_[M+OAC]1-		
648.6300	Cer(42:1)_[M-H]1-		
682.5910	Cer(42:2)_[M+Cl]1-		
646.6144	Cer(42:2)_[M-H]1-		
1401.9807	CL(68:3)_[M-H]1-		
1399.9650	CL(68:4)_[M-H]1-		
1436.0589	CL(70:0)_[M-H]1-		
717.5253	CL(70:01)_[M]2-		
715.5096	CL(70:03)_[M]2-		
713.4940	CL(70:05)_[M]2-		
1434.0433	CL(70:1)_[M-H]1-		
1430.0120	CL(70:3)_[M-H]1-		
1427.9963	CL(70:4)_[M-H]1-		
1425.9807	CL(70:5)_[M-H]1-		
1423.9650	CL(70:6)_[M-H]1-		
1450.0651	CL(71:0)_[M-H]1-		
1448.0495	CL(71:1)_[M-H]1-		
1464.0902	CL(72:0)_[M-H]1-		
1462.0746	CL(72:1)_[M-H]1-		
1458.0433	CL(72:3)_[M-H]1-		
1456.0276	CL(72:4)_[M-H]1-		
1454.0120	CL(72:5)_[M-H]1-		
1451.9963	CL(72:6)_[M-H]1-		
1449.9807	CL(72:7)_[M-H]1-		
1447.9650	CL(72:8)_[M-H]1-		
1474.0651	CL(73:2)_[M-H]1-		
1472.0495	CL(73:3)_[M-H]1-		
1470.0338	CL(73:4)_[M-H]1-		
1468.0182	CL(73:5)_[M-H]1-		
1492.1215	CL(74:0)_[M-H]1-		
743.5409	CL(74:03)_[M]2-		
1490.1059	CL(74:1)_[M-H]1-		
1471.9650	CL(74:10)_[M-H]1-		
1488.0902	CL(74:2)_[M-H]1-		
1484.0589	CL(74:4)_[M-H]1-		
1482.0433	CL(74:5)_[M-H]1-		
1480.0276	CL(74:6)_[M-H]1-		
1478.0120	CL(74:7)_[M-H]1-		
1475.9963	CL(74:8)_[M-H]1-		
1473.9807	CL(74:9)_[M-H]1-		
1502.0964	CL(75:2)_[M-H]1-		
1494.0338	CL(75:6)_[M-H]1-		
1492.0182	CL(75:7)_[M-H]1-		
1490.0025	CL(75:8)_[M-H]1-		
1520.1528	CL(76:0)_[M-H]1-		
753.5253	CL(76:07)_[M]2-		
1499.9963	CL(76:10)_[M-H]1-		

1497.9807	CL(76:11)_[M-H]1-	
1495.9650	CL(76:12)_[M-H]1-	
1516.1215	CL(76:2)_[M-H]1-	
1514.1059	CL(76:3)_[M-H]1-	
1504.0276	CL(76:8)_[M-H]1-	
1502.0120	CL(76:9)_[M-H]1-	
1530.1277	CL(77:2)_[M-H]1-	
1518.0338	CL(77:8)_[M-H]1-	
1516.0182	CL(77:9)_[M-H]1-	
774.5957	CL(78:00)_[M]2-	
768.5487	CL(78:06)_[M]2-	
1528.0276	CL(78:10)_[M-H]1-	
1523.9963	CL(78:12)_[M-H]1-	
1521.9807	CL(78:13)_[M-H]1-	
1519.9650	CL(78:14)_[M-H]1-	
1544.1528	CL(78:2)_[M-H]1-	
1530.0433	CL(78:9)_[M-H]1-	
1538.0025	CL(79:12)_[M-H]1-	
675.5331	LPA(35:0)_[M]-	
540.3307	LPC(15:0)_[M+OAC]1-	
566.3463	LPC(17:1)_[M+OAC]1-	LPS(21:0)_[M-H]1-
554.3019	LPC(18:2)_[M+Cl]1-	
578.3463	LPC(18:2)_[M+OAC]1-	LPS(22:1)_[M-H]1-
590.3463	LPC(19:3)_[M+OAC]1-	
604.3620	LPC(20:3)_[M+OAC]1-	LPS(24:2)_[M-H]1-
578.3019	LPC(20:4)_[M+Cl]1-	
602.3463	LPC(20:4)_[M+OAC]1-	LPS(24:3)_[M-H]1-
612.3801	LPC(22:1)_[M+Cl]1-	
610.3645	LPC(22:2)_[M+Cl]1-	
634.4089	LPC(22:2)_[M+OAC]1-	LPS(26:1)_[M-H]1-
602.3019	LPC(22:6)_[M+Cl]1-	
626.3463	LPC(22:6)_[M+OAC]1-	
666.4715	LPC(24:0)_[M+OAC]1-	
454.2575	LPC(O-10:1)_[M+OAC]1-	
570.3696	LPC-2O(20:1)_[M+Cl]1-	
628.4478	LPC-2O(24:0)_[M+Cl]1-	
542.3383	LPC-O_18:1)_[M+Cl]1-	
410.2313	LPE(13:0)_[M-H]1-	
422.2313	LPE(14:1)_[M-H]1-	
452.2783	LPE(16:0)_[M-H]1-	
480.3096	LPE(18:0)_[M-H]1-	
478.2939	LPE(18:1)_[M-H]1-	
508.3409	LPE(20:0)_[M-H]1-	
506.3252	LPE(20:1)_[M-H]1-	
504.3096	LPE(20:2)_[M-H]1-	
500.2783	LPE(20:4)_[M-H]1-	
528.3096	LPE(22:4)_[M-H]1-	
524.2783	LPE(22:6)_[M-H]1-	
436.2833	LPE-P(16:0)_[M-H]1-	
619.2889	LPI(20:4)_[M-H]1-	
645.4495	PA(32:1)_[M-H]1-	
673.4808	PA(34:1)_[M-H]1-	TG_oxid(35:1)_[M+Cl]1-
671.4652	PA(34:2)_[M-H]1-	TG_oxid(35:2)_[M+Cl]1-
687.4965	PA(35:1)_[M-H]1-	TG_oxid(36:1)_[M+Cl]1-
703.5278	PA(36:0)_[M-H]1-	TG_oxid(37:0)_[M+Cl]1-
701.5121	PA(36:1)_[M-H]1-	TG_oxid(37:1)_[M+Cl]1-
699.4965	PA(36:2)_[M-H]1-	TG_oxid(37:2)_[M+Cl]1-
695.4652	PA(36:4)_[M-H]1-	
705.4495	PA(37:6)_[M-H]1-	
721.4808	PA(38:5)_[M-H]1-	
719.4652	PA(38:6)_[M-H]1-	
594.3413	PC(18:1)_[M+OAC]1-	PS(23:0)_[M-H]1-
584.3125	PC(19:1)_[M+Cl]1-	

608.3569	PC(19:1)_[M+OAC]1-		
636.3882	PC(21:1)_[M+OAC]1-	PS(25:0)_[M-H]1-	
734.4978	PC(28:1)_[M+OAC]1-	PS(32:0)_[M-H]1-	
732.4821	PC(28:2)_[M+OAC]1-	PS(32:1)_[M-H]1-	
758.4978	PC(30:3)_[M+OAC]1-	PS(34:2)_[M-H]1-	
778.5604	PC(31:0)_[M+OAC]1-		
786.5291	PC(32:3)_[M+OAC]1-	PS(36:2)_[M-H]1-	
784.5134	PC(32:4)_[M+OAC]1-	PS(36:3)_[M-H]1-	
782.4978	PC(32:5)_[M+OAC]1-	PS(36:4)_[M-H]1-	
778.5159	PC(33:2)_[M+Cl]1-		
802.5604	PC(33:2)_[M+OAC]1-	PS(37:1)_[M-H]1-	
800.5447	PC(33:3)_[M+OAC]1-	PS(37:2)_[M-H]1-	
798.5291	PC(33:4)_[M+OAC]1-	PS(37:3)_[M-H]1-	
794.5472	PC(34:1)_[M+Cl]1-		
818.5917	PC(34:1)_[M+OAC]1-	PS(38:0)_[M-H]1-	
792.5316	PC(34:2)_[M+Cl]1-		
816.5760	PC(34:2)_[M+OAC]1-	PS(38:1)_[M-H]1-	
814.5604	PC(34:3)_[M+OAC]1-	PS(38:2)_[M-H]1-	
812.5447	PC(34:4)_[M+OAC]1-	PS(38:3)_[M-H]1-	
810.5291	PC(34:5)_[M+OAC]1-	PS(38:4)_[M-H]1-	
808.5134	PC(34:6)_[M+OAC]1-	PS(38:5)_[M-H]1-	
808.5629	PC(35:1)_[M+Cl]1-		
828.5760	PC(35:3)_[M+OAC]1-	PS(39:2)_[M-H]1-	
800.5003	PC(35:5)_[M+Cl]1-		
822.5291	PC(35:6)_[M+OAC]1-	PS(39:5)_[M-H]1-	
848.6386	PC(36:0)_[M+OAC]1-		
846.6230	PC(36:1)_[M+OAC]1-	PS(40:0)_[M-H]1-	
820.5629	PC(36:2)_[M+Cl]1-		
844.6073	PC(36:2)_[M+OAC]1-	PS(40:1)_[M-H]1-	
842.5917	PC(36:3)_[M+OAC]1-	PS(40:2)_[M-H]1-	
816.5316	PC(36:4)_[M+Cl]1-		
840.5760	PC(36:4)_[M+OAC]1-	PS(40:3)_[M-H]1-	
838.5604	PC(36:5)_[M+OAC]1-	PS(40:4)_[M-H]1-	
834.5291	PC(36:7)_[M+OAC]1-	PS(40:6)_[M-H]1-	
808.4690	PC(36:8)_[M+Cl]1-		
832.5134	PC(36:8)_[M+OAC]1-	PS(40:7)_[M-H]1-	
860.6386	PC(37:1)_[M+OAC]1-	PS(41:0)_[M-H]1-	
858.6230	PC(37:2)_[M+OAC]1-	PS(41:1)_[M-H]1-	
852.5760	PC(37:5)_[M+OAC]1-	PS(41:4)_[M-H]1-	
850.5604	PC(37:6)_[M+OAC]1-	PS(41:5)_[M-H]1-	
824.5003	PC(37:7)_[M+Cl]1-		
848.5447	PC(37:7)_[M+OAC]1-	PS(41:6)_[M-H]1-	
852.6255	PC(38:0)_[M+Cl]1-		
876.6699	PC(38:0)_[M+OAC]1-		
850.6098	PC(38:1)_[M+Cl]1-	PC-O(38:6)_[M+OAC]1-	PS-P(42:1)_[M-H]1-
874.6543	PC(38:1)_[M+OAC]1-	PS(42:0)_[M-H]1-	
848.5942	PC(38:2)_[M+Cl]1-		
872.6386	PC(38:2)_[M+OAC]1-	PS(42:1)_[M-H]1-	
870.6230	PC(38:3)_[M+OAC]1-	PS(42:2)_[M-H]1-	
844.5629	PC(38:4)_[M+Cl]1-		
868.6073	PC(38:4)_[M+OAC]1-	PS(42:3)_[M-H]1-	
866.5917	PC(38:5)_[M+OAC]1-	PS(42:4)_[M-H]1-	
840.5316	PC(38:6)_[M+Cl]1-		
864.5760	PC(38:6)_[M+OAC]1-	PS(42:5)_[M-H]1-	
862.5604	PC(38:7)_[M+OAC]1-	PS(42:6)_[M-H]1-	
858.5291	PC(38:9)_[M+OAC]1-	PS(42:8)_[M-H]1-	
866.6411	PC(39:0)_[M+Cl]1-		
890.6856	PC(39:0)_[M+OAC]1-		
864.6255	PC(39:1)_[M+Cl]1-		
888.6699	PC(39:1)_[M+OAC]1-	PS(43:0)_[M-H]1-	
862.6098	PC(39:2)_[M+Cl]1-		
886.6543	PC(39:2)_[M+OAC]1-	PS(43:1)_[M-H]1-	
860.5942	PC(39:3)_[M+Cl]1-		

858.5785	PC(39:4)_[M+Cl]1-	
882.6230	PC(39:4)_[M+OAC]1-	PS(43:3)_[M-H]1-
880.6073	PC(39:5)_[M+OAC]1-	PS(43:4)_[M-H]1-
854.5472	PC(39:6)_[M+Cl]1-	
878.5917	PC(39:6)_[M+OAC]1-	PS(43:5)_[M-H]1-
876.5760	PC(39:7)_[M+OAC]1-	PS(43:6)_[M-H]1-
850.5159	PC(39:8)_[M+Cl]1-	
874.5604	PC(39:8)_[M+OAC]1-	PS(43:7)_[M-H]1-
880.6568	PC(40:0)_[M+Cl]1-	
904.7012	PC(40:0)_[M+OAC]1-	
878.6411	PC(40:1)_[M+Cl]1-	
902.6856	PC(40:1)_[M+OAC]1-	PS(44:0)_[M-H]1-
884.5447	PC(40:10)_[M+OAC]1	PS(44:9)_[M-H]1-
876.6255	PC(40:2)_[M+Cl]1-	
900.6699	PC(40:2)_[M+OAC]1-	PS(44:1)_[M-H]1-
874.6098	PC(40:3)_[M+Cl]1-	
898.6543	PC(40:3)_[M+OAC]1-	PS(44:2)_[M-H]1-
872.5942	PC(40:4)_[M+Cl]1-	
896.6386	PC(40:4)_[M+OAC]1-	PS(44:3)_[M-H]1-
894.6230	PC(40:5)_[M+OAC]1-	PS(44:4)_[M-H]1-
868.5629	PC(40:6)_[M+Cl]1-	
892.6073	PC(40:6)_[M+OAC]1-	PS(44:5)_[M-H]1-
866.5472	PC(40:7)_[M+Cl]1-	
890.5917	PC(40:7)_[M+OAC]1-	PS(44:6)_[M-H]1-
864.5316	PC(40:8)_[M+Cl]1-	
862.5159	PC(40:9)_[M+Cl]1-	
886.5604	PC(40:9)_[M+OAC]1-	PS(44:8)_[M-H]1-
918.7169	PC(41:0)_[M+OAC]1-	
916.7012	PC(41:1)_[M+OAC]1-	
890.6411	PC(41:2)_[M+Cl]1-	
914.6856	PC(41:2)_[M+OAC]1-	
888.6255	PC(41:3)_[M+Cl]1-	
886.6098	PC(41:4)_[M+Cl]1-	
910.6543	PC(41:4)_[M+OAC]1-	
884.5942	PC(41:5)_[M+Cl]1-	
908.6386	PC(41:5)_[M+OAC]1-	
882.5785	PC(41:6)_[M+Cl]1-	
906.6230	PC(41:6)_[M+OAC]1-	
904.6073	PC(41:7)_[M+OAC]1-	
932.7325	PC(42:0)_[M+OAC]1-	
930.7169	PC(42:1)_[M+OAC]1-	
912.5760	PC(42:10)_[M+OAC]1-	
910.5604	PC(42:11)_[M+OAC]1-	
904.6568	PC(42:2)_[M+Cl]1-	
928.7012	PC(42:2)_[M+OAC]1-	
902.6411	PC(42:3)_[M+Cl]1-	
900.6255	PC(42:4)_[M+Cl]1-	
924.6699	PC(42:4)_[M+OAC]1-	
898.6098	PC(42:5)_[M+Cl]1-	
922.6543	PC(42:5)_[M+OAC]1-	
896.5942	PC(42:6)_[M+Cl]1-	
920.6386	PC(42:6)_[M+OAC]1-	
894.5785	PC(42:7)_[M+Cl]1-	
946.7482	PC(43:0)_[M+OAC]1-	
944.7325	PC(43:1)_[M+OAC]1-	
918.6724	PC(43:2)_[M+Cl]1-	
942.7169	PC(43:2)_[M+OAC]1-	
914.6411	PC(43:4)_[M+Cl]1-	
938.6856	PC(43:4)_[M+OAC]1-	
910.6098	PC(43:6)_[M+Cl]1-	
934.6543	PC(43:6)_[M+OAC]1-	
958.7482	PC(44:1)_[M+OAC]1-	
940.6073	PC(44:10)_[M+OAC]1-	

912.5316	PC(44:12)_[M+Cl]1-	
936.5760	PC(44:12)_[M+OAC]1-	
956.7325	PC(44:2)_[M+OAC]1-	
930.6724	PC(44:3)_[M+Cl]1-	
928.6568	PC(44:4)_[M+Cl]1-	
952.7012	PC(44:4)_[M+OAC]1-	
926.6411	PC(44:5)_[M+Cl]1-	
950.6856	PC(44:5)_[M+OAC]1-	
924.6255	PC(44:6)_[M+Cl]1-	
948.6699	PC(44:6)_[M+OAC]1-	
922.6098	PC(44:7)_[M+Cl]1-	
920.5942	PC(44:8)_[M+Cl]1-	
988.7951	PC(46:0)_[M+OAC]1-	
986.7795	PC(46:1)_[M+OAC]1-	
984.7638	PC(46:2)_[M+OAC]1-	
1016.8264	PC(48:0)_[M+OAC]1-	
1012.7951	PC(48:1)_[M+OAC]1-	
1044.8577	PC(50:0)_[M+OAC]1-	
1048.8446	PC(52:0)_[M+Cl]1-	
1040.7820	PC(52:4)_[M+Cl]1-	
1064.8264	PC(52:4)_[M+OAC]1-	
684.5104	PC-2O(28:0)_[M+Cl]1-	
740.5730	PC-2O(32:0)_[M+Cl]1-	
768.6043	PC-2O(34:0)_[M+Cl]1-	
816.6488	PC-2O(36:1)_[M+OAC]1-	PE(41:0)_[M-H]1-
852.6982	PC-2O(40:0)_[M+Cl]1-	
876.7427	PC-2O(40:0)_[M+OAC]1-	
554.3463	PC-O(16:0)_[M+OAC]1-	
568.3620	PC-O(17:0)_[M+OAC]1-	
558.3332	PC-O(18:0)_[M+Cl]1-	
582.3776	PC-O(18:0)_[M+OAC]1-	
556.3175	PC-O(18:1)_[M+Cl]1-	
594.3776	PC-O(19:1)_[M+OAC]1-	
610.4089	PC-O(20:0)_[M+OAC]1-	
624.4246	PC-O(21:0)_[M+OAC]1-	
638.4402	PC-O(22:0)_[M+OAC]1-	
790.5967	PC-O(33:1)_[M+OAC]1-	PS-O(37:0)_[M-H]1-
788.5811	PC-O(33:2)_[M+OAC]1-	PS-O(37:1)_[M-H]1-
798.5654	PC-O(34:4)_[M+OAC]1-	PS-O(38:3)_[M-H]1-
818.6280	PC-O(35:1)_[M+OAC]1-	PS-O(39:0)_[M-H]1-
816.6124	PC-O(35:2)_[M+OAC]1-	PS-O(39:1)_[M-H]1-
812.5811	PC-O(35:4)_[M+OAC]1-	PS-O(39:3)_[M-H]1-
808.5992	PC-O(36:1)_[M+Cl]1-	
806.5836	PC-O(36:2)_[M+Cl]1-	
830.6280	PC-O(36:2)_[M+OAC]1-	PS-O(40:1)_[M-H]1-
804.5679	PC-O(36:3)_[M+Cl]1-	
828.6124	PC-O(36:3)_[M+OAC]1-	PS-O(40:2)_[M-H]1-
826.5967	PC-O(36:4)_[M+OAC]1-	PS-O(40:3)_[M-H]1-
824.5811	PC-O(36:5)_[M+OAC]1-	PS-O(40:4)_[M-H]1-
846.6593	PC-O(37:1)_[M+OAC]1-	PS-O(41:0)_[M-H]1-
844.6437	PC-O(37:2)_[M+OAC]1-	PS-O(41:1)_[M-H]1-
840.6124	PC-O(37:4)_[M+OAC]1-	
838.6462	PC-O(38:0)_[M+Cl]1-	
862.6906	PC-O(38:0)_[M+OAC]1-	
836.6305	PC-O(38:1)_[M+Cl]1-	
834.6149	PC-O(38:2)_[M+Cl]1-	
832.5992	PC-O(38:3)_[M+Cl]1-	
856.6437	PC-O(38:3)_[M+OAC]1-	PS-O(42:2)_[M-H]1-
830.5836	PC-O(38:4)_[M+Cl]1-	
826.5523	PC-O(38:6)_[M+Cl]1-	
876.7063	PC-O(39:0)_[M+OAC]1-	
874.6906	PC-O(39:1)_[M+OAC]1-	
890.7219	PC-O(40:0)_[M+OAC]1-	

888.7063	PC-O(40:1)_[M+OAC]1-	
862.6543	PC-O(40:2)_[M+Cl]1-	
860.6305	PC-O(40:3)_[M+Cl]1-	
858.6149	PC-O(40:4)_[M+Cl]1-	
882.6593	PC-O(40:4)_[M+OAC]1-	
856.5992	PC-O(40:5)_[M+Cl]1-	
880.6437	PC-O(40:5)_[M+OAC]1-	
854.5836	PC-O(40:6)_[M+Cl]1-	
904.7376	PC-O(41:0)_[M+OAC]1-	
918.7532	PC-O(42:0)_[M+OAC]1-	
892.6931	PC-O(42:1)_[M+Cl]1-	
916.7376	PC-O(42:1)_[M+OAC]1-	
890.6775	PC-O(42:2)_[M+Cl]1-	
914.7219	PC-O(42:2)_[M+OAC]1-	
888.6618	PC-O(42:3)_[M+Cl]1-	
886.6462	PC-O(42:4)_[M+Cl]1-	
882.6149	PC-O(42:6)_[M+Cl]1-	
916.6931	PC-O(44:3)_[M+Cl]1-	
940.7376	PC-O(44:3)_[M+OAC]1-	
914.6775	PC-O(44:4)_[M+Cl]1-	
912.6618	PC-O(44:5)_[M+Cl]1-	
764.5811	PC-O(31:0)_[M+OAC]1-	
762.5654	PC-O(31:1)_[M+OAC]1-	PS-O(35:0)_[M-H]1-
720.5185	PC-P(28:0)_[M+OAC]1-	PS-O(32:0)_[M-H]1-
734.5341	PC-P(29:0)_[M+OAC]1-	PS-O(33:0)_[M-H]1-
786.5654	PC-P(33:2)_[M+OAC]1-	PS-O(37:2)_[M-H]1-
796.5498	PC-P(34:4)_[M+OAC]1-	PS-O(38:4)_[M-H]1-
822.5654	PC-P(36:5)_[M+OAC]1-	PS-O(40:5)_[M-H]1-
842.6280	PC-P(37:2)_[M+OAC]1-	
824.5366	PC-P(38:6)_[M+Cl]1-	
878.6775	PC-P(41:0)_[M+Cl]1-	
908.6750	PC-P(42:4)_[M+OAC]1-	
1033.7347	PE(34:1)-15-isoLG pyrrole	
648.4610	PE(29:0)_[M-H]1-	
676.4923	PE(31:0)_[M-H]1-	
674.4766	PE(31:1)_[M-H]1-	
672.4610	PE(31:2)_[M-H]1-	
668.4297	PE(31:4)_[M-H]1-	
690.5079	PE(32:0)_[M-H]1-	
688.4923	PE(32:1)_[M-H]1-	
704.5236	PE(33:0)_[M-H]1-	
696.4610	PE(33:4)_[M-H]1-	
718.5392	PE(34:0)_[M-H]1-	
716.5236	PE(34:1)_[M-H]1-	
714.5079	PE(34:2)_[M-H]1-	
732.5549	PE(35:0)_[M-H]1-	
730.5392	PE(35:1)_[M-H]1-	
726.5079	PE(35:3)_[M-H]1-	
720.4610	PE(35:6)_[M-H]1-	
744.5549	PE(36:1)_[M-H]1-	
742.5392	PE(36:2)_[M-H]1-	
738.5079	PE(36:4)_[M-H]1-	
736.4923	PE(36:5)_[M-H]1-	
734.4766	PE(36:6)_[M-H]1-	
760.5862	PE(37:0)_[M-H]1-	
758.5705	PE(37:1)_[M-H]1-	
756.5549	PE(37:2)_[M-H]1-	
772.5862	PE(38:1)_[M-H]1-	
770.5705	PE(38:2)_[M-H]1-	
766.5392	PE(38:4)_[M-H]1-	
764.5236	PE(38:5)_[M-H]1-	
762.5079	PE(38:6)_[M-H]1-	
788.6175	PE(39:0)_[M-H]1-	

786.6018	PE(39:1)_[M-H]1-
784.5862	PE(39:2)_[M-H]1-
782.5705	PE(39:3)_[M-H]1-
780.5549	PE(39:4)_[M-H]1-
776.5236	PE(39:6)_[M-H]1-
802.6331	PE(40:0)_[M-H]1-
800.6175	PE(40:1)_[M-H]1-
798.6018	PE(40:2)_[M-H]1-
794.5705	PE(40:4)_[M-H]1-
790.5392	PE(40:6)_[M-H]1-
788.5236	PE(40:7)_[M-H]1-
784.4923	PE(40:9)_[M-H]1-
808.5862	PE(41:4)_[M-H]1-
828.6488	PE(42:1)_[M-H]1-
826.6331	PE(42:2)_[M-H]1-
824.6175	PE(42:3)_[M-H]1-
818.5705	PE(42:6)_[M-H]1-
814.5392	PE(42:8)_[M-H]1-
840.6488	PE(43:2)_[M-H]1-
854.6644	PE(44:2)_[M-H]1-
852.6488	PE(44:3)_[M-H]1-
850.6331	PE(44:4)_[M-H]1-
884.7114	PE(46:1)_[M-H]1-
718.6120	PE-2O(36:0)_[M-H]1-
660.4974	PE-O(31:1)_[M-H]1-
676.5287	PE-O(32:0)_[M-H]1-
674.5130	PE-O(32:1)_[M-H]1-
704.5600	PE-O(34:0)_[M-H]1-
702.5443	PE-O(34:1)_[M-H]1-
700.5287	PE-O(34:2)_[M-H]1-
698.5130	PE-O(34:3)_[M-H]1-
714.5443	PE-O(35:2)_[M-H]1-
732.5913	PE-O(36:0)_[M-H]1-
728.5600	PE-O(36:2)_[M-H]1-
726.5443	PE-O(36:3)_[M-H]1-
724.5287	PE-O(36:4)_[M-H]1-
722.5130	PE-O(36:5)_[M-H]1-
742.5756	PE-O(37:2)_[M-H]1-
760.6226	PE-O(38:0)_[M-H]1-
758.6069	PE-O(38:1)_[M-H]1-
756.5913	PE-O(38:2)_[M-H]1-
754.5756	PE-O(38:3)_[M-H]1-
752.5600	PE-O(38:4)_[M-H]1-
750.5443	PE-O(38:5)_[M-H]1-
748.5287	PE-O(38:6)_[M-H]1-
786.6382	PE-O(40:1)_[M-H]1-
784.6226	PE-O(40:2)_[M-H]1-
782.6069	PE-O(40:3)_[M-H]1-
776.5600	PE-O(40:6)_[M-H]1-
802.6695	PE-O(41:0)_[M-H]1-
816.6852	PE-O(42:0)_[M-H]1-
814.6695	PE-O(42:1)_[M-H]1-
672.4974	PE-P(32:1)_[M-H]1-
720.4974	PE-P(36:5)_[M-H]1-
746.5130	PE-P(38:6)_[M-H]1-
770.6069	PE-P(39:1)_[M-H]1-
774.5443	PE-P(40:6)_[M-H]1-
772.5287	PE-P(40:7)_[M-H]1-
637.4081	PG(26:0)_[M-H]1-
721.5020	PG(32:0)_[M-H]1-
733.5020	PG(33:1)_[M-H]1-
749.5333	PG(34:0)_[M-H]1-
747.5176	PG(34:1)_[M-H]1-

745.5020	PG(34:2)_[M-H]1-	
743.4863	PG(34:3)_[M-H]1-	
741.4707	PG(34:4)_[M-H]1-	
759.5176	PG(35:2)_[M-H]1-	
757.5020	PG(35:3)_[M-H]1-	
777.5646	PG(36:0)_[M-H]1-	
775.5489	PG(36:1)_[M-H]1-	
773.5333	PG(36:2)_[M-H]1-	
771.5176	PG(36:3)_[M-H]1-	
769.5020	PG(36:4)_[M-H]1-	
793.5020	PG(38:6)_[M-H]1-	
749.5702	PG-O(35:0)_[M-H]1-	
815.6171	PG-O(40:2)_[M-H]1-	
833.6641	PG-O(41:0)_[M-H]1-	
835.5858	PG-O(42:6)_[M-H]1-	
828.4918	PGP(34:1)_[M-H]1-	
833.5702	PG-P(42:6)_[M-H]1-	
767.4711	PI(29:0)_[M-H]1-	
791.4711	PI(31:2)_[M-H]1-	
807.5024	PI(32:1)_[M-H]1-	
823.5337	PI(33:0)_[M-H]1-	PA(46:10)_[M-H]1-
835.5337	PI(34:1)_[M-H]1-	
831.5024	PI(34:3)_[M-H]1-	
851.5650	PI(35:0)_[M-H]1-	PA(48:10)_[M-H]1-
847.5337	PI(35:2)_[M-H]1-	
863.5650	PI(36:1)_[M-H]1-	
855.5024	PI(36:5)_[M-H]1-	
879.5963	PI(37:0)_[M-H]1-	PA(50:10)_[M-H]1-
875.5650	PI(37:2)_[M-H]1-	
871.5337	PI(37:4)_[M-H]1-	
867.5024	PI(37:6)_[M-H]1-	
893.6119	PI(38:0)_[M-H]1-	
891.5963	PI(38:1)_[M-H]1-	
883.5337	PI(38:5)_[M-H]1-	
907.6276	PI(39:0)_[M-H]1-	PA(52:10)_[M-H]1-
903.5963	PI(39:2)_[M-H]1-	
899.5650	PI(39:4)_[M-H]1-	
895.5337	PI(39:6)_[M-H]1-	
911.5650	PI(40:5)_[M-H]1-	
907.5337	PI(40:7)_[M-H]1-	
927.5963	PI(41:4)_[M-H]1-	
925.5806	PI(41:5)_[M-H]1-	
939.5963	PI(42:5)_[M-H]1-	
935.5650	PI(42:7)_[M-H]1-	
957.6432	PI(43:3)_[M-H]1-	
955.6276	PI(43:4)_[M-H]1-	
953.6119	PI(43:5)_[M-H]1-	
971.5650	PI(45:10)_[M-H]1-	
979.6276	PI(45:6)_[M-H]1-	
973.5806	PI(45:9)_[M-H]1-	
991.6276	PI(46:7)_[M-H]1-	
987.5963	PI(46:9)_[M-H]1-	
1001.6119	PI(47:9)_[M-H]1-	
1013.6119	PI(48:10)_[M-H]1-	
1015.6276	PI(48:9)_[M-H]1-	
1029.6432	PI(49:9)_[M-H]1-	
1045.6745	PI(50:8)_[M-H]1-	
1043.6589	PI(50:9)_[M-H]1-	
1069.7684	PI(51:3)_[M-H]1-	
1071.6902	PI(52:9)_[M-H]1-	
767.5080	PI-O(30:0)_[M-H]1-	
779.5080	PI-O(31:1)_[M-H]1-	
807.5393	PI-O(33:1)_[M-H]1-	

823.5706	PI-O(34:0)_[M-H]1-
837.5862	PI-O(35:0)_[M-H]1-
835.5706	PI-O(35:1)_[M-H]1-
833.5549	PI-O(35:2)_[M-H]1-
843.5393	PI-O(36:4)_[M-H]1-
865.6175	PI-O(37:0)_[M-H]1-
863.6019	PI-O(37:1)_[M-H]1-
861.5862	PI-O(37:2)_[M-H]1-
877.6175	PI-O(38:1)_[M-H]1-
893.6488	PI-O(39:0)_[M-H]1-
891.6332	PI-O(39:1)_[M-H]1-
903.6332	PI-O(40:2)_[M-H]1-
921.6801	PI-O(41:0)_[M-H]1-
933.6801	PI-O(42:1)_[M-H]1-
927.6332	PI-O(42:4)_[M-H]1-
664.4195	PS(27:0)_[M-H]1-
692.4508	PS(29:0)_[M-H]1-
704.4508	PS(30:1)_[M-H]1-
780.4821	PS(36:5)_[M-H]1-
806.4978	PS(38:6)_[M-H]1-
804.4821	PS(38:7)_[M-H]1-
830.4978	PS(40:8)_[M-H]1-
828.4821	PS(40:9)_[M-H]1-
854.4978	PS(42:10)_[M-H]1-
852.4821	PS(42:11)_[M-H]1-
856.5134	PS(42:9)_[M-H]1-
882.5291	PS(44:10)_[M-H]1-
678.4715	PS-O(29:0)_[M-H]1-
664.4559	PS-O(29:1)_[M-H]1-
692.4872	PS-O(30:0)_[M-H]1-
690.4715	PS-O(30:1)_[M-H]1-
732.5185	PS-O(33:1)_[M-H]1-
758.5341	PS-O(35:2)_[M-H]1-
676.4559	PS-P(29:0)_[M-H]1-
688.4559	PS-P(30:1)_[M-H]1-
784.5498	PS-P(37:2)_[M-H]1-
749.5814	SM(33:0)_[M+OAC]1-
747.5658	SM(33:1)_[M+OAC]1-
745.5501	SM(33:2)_[M+OAC]1-
763.5971	SM(34:0)_[M+OAC]1-
761.5814	SM(34:1)_[M+OAC]1-
759.5658	SM(34:2)_[M+OAC]1-
777.6127	SM(35:0)_[M+OAC]1-
775.5971	SM(35:1)_[M+OAC]1-
791.6284	SM(36:0)_[M+OAC]1-
789.6127	SM(36:1)_[M+OAC]1-
787.5971	SM(36:2)_[M+OAC]1-
803.6284	SM(37:1)_[M+OAC]1-
819.6597	SM(38:0)_[M+OAC]1-
817.6440	SM(38:1)_[M+OAC]1-
815.6284	SM(38:2)_[M+OAC]1-
831.6597	SM(39:1)_[M+OAC]1-
829.6440	SM(39:2)_[M+OAC]1-
845.6753	SM(40:1)_[M+OAC]1-
843.6597	SM(40:2)_[M+OAC]1-
859.6910	SM(41:0)_[M+OAC]1-
857.6753	SM(41:1)_[M+OAC]1-
875.7223	SM(42:0)_[M+OAC]1-
873.7066	SM(42:1)_[M+OAC]1-
871.6910	SM(42:2)_[M+OAC]1-
869.6753	SM(42:3)_[M+OAC]1-
903.7536	SM(44:0)_[M+OAC]1-
901.7379	SM(44:1)_[M+OAC]1-

Positive ionisation mode			
m/z	Lipid variable	Isobar 1	Isobar 2
383.367	Campesterol_[M+H-H2O]1+		
639.608	Campesteryl ester (16:0)		
667.639	Campesteryl ester (18:0)		
693.654	Campesteryl ester (20:1)		
713.621	Campesteryl ester (20:2)_[M+Na]1+		
743.668	Campesteryl ester (22:1)_[M+Na]1+		
736.697	Campesteryl ester (22:2)_[M+NH4]1+		
673.589	CE(18:01)_[M+Na]1+		
671.574	CE(18:02)_[M+Na]1+		
701.621	CE(20:01)_[M+Na]1+		
697.589	CE(20:03)_[M+Na]1+		
695.574	CE(20:04)_[M+Na]1+		
690.618	CE(20:04)_[M+NH4]1+		
725.621	CE(22:03)_[M+Na]1+		
721.589	CE(22:05)_[M+Na]1+		
716.634	CE(22:05)_[M+NH4]1+		
719.574	CE(22:06)_[M+Na]1+		
714.618	CE(22:06)_[M+NH4]1+	TG_oxid(39:00)	
754.744	CE(24:00)_[M+NH4]1+		
742.707	CE_oxid(22:00)		
412.379	Cer(25:01)		
440.410	Cer(27:01)		
454.426	Cer(28:01)		
468.441	Cer(29:01)		
536.504	Cer(34:02)		
534.525	Cer(35:01)_[M+H-H2O]1+		
532.509	Cer(35:02)_[M+H-H2O]1+		
548.540	Cer(36:01)_[M+H-H2O]1+		
564.535	Cer(36:02)		
546.525	Cer(36:02)_[M+H-H2O]1+		
594.582	Cer(38:01)		
576.571	Cer(38:01)_[M+H-H2O]1+		
592.566	Cer(38:02)		
590.587	Cer(39:01)_[M+H-H2O]1+		
588.571	Cer(39:02)_[M+H-H2O]1+		
622.613	Cer(40:01)		
604.603	Cer(40:01)_[M+H-H2O]1+		
620.598	Cer(40:02)		
618.582	Cer(40:03)		
618.618	Cer(41:01)_[M+H-H2O]1+		
634.613	Cer(41:02)		
652.660	Cer(42:00)		
650.645	Cer(42:01)		
632.634	Cer(42:01)_[M+H-H2O]1+		
648.629	Cer(42:02)		
630.618	Cer(42:02)_[M+H-H2O]1+		
646.613	Cer(42:03)		
660.665	Cer(44:01)_[M+H-H2O]1+		
658.650	Cer(44:02)_[M+H-H2O]1+		
674.645	Cer(44:03)		
706.707	Cer(46:01)		
369.352	Cholesterol)_[M+H-H2O]1+	lathosterol_[M+H-H2O]1+	
383.316	DG(20:00)_[M+H-H2O]1+		
411.347	DG(22:00)_[M+H-H2O]1+		
467.410	DG(26:00)_[M+H-H2O]1+		
465.394	DG(26:01)_[M+H-H2O]1+		
463.379	DG(26:02)_[M+H-H2O]1+		
493.425	DG(28:01)_[M+H-H2O]1+		
491.410	DG(28:02)_[M+H-H2O]1+		

507.441	DG(29:01)_[M+H-H2O]1+	
505.425	DG(29:02)_[M+H-H2O]1+	
519.441	DG(30:02)_[M+H-H2O]1+	
533.456	DG(31:02)_[M+H-H2O]1+	
531.441	DG(31:03)_[M+H-H2O]1+	
547.472	DG(32:02)_[M+H-H2O]1+	
545.456	DG(32:03)_[M+H-H2O]1+	
543.441	DG(32:04)_[M+H-H2O]1+	
563.503	DG(33:01)_[M+H-H2O]1+	
561.488	DG(33:02)_[M+H-H2O]1+	
575.503	DG(34:02)_[M+H-H2O]1+	
610.541	DG(34:02)_[M+NH4]1+	
573.488	DG(34:03)_[M+H-H2O]1+	
591.535	DG(35:01)_[M+H-H2O]1+	
589.520	DG(35:02)_[M+H-H2O]1+	
585.488	DG(35:04)_[M+H-H2O]1+	
583.473	DG(35:05)_[M+H-H2O]1+	
605.551	DG(36:01)_[M+H-H2O]1+	
640.588	DG(36:01)_[M+NH4]1+	
603.535	DG(36:02)_[M+H-H2O]1+	
601.520	DG(36:03)_[M+H-H2O]1+	
599.504	DG(36:04)_[M+H-H2O]1+	
597.488	DG(36:05)_[M+H-H2O]1+	
619.567	DG(37:01)_[M+H-H2O]1+	
615.535	DG(37:03)_[M+H-H2O]1+	
650.572	DG(37:03)_[M+NH4]1+	
613.520	DG(37:04)_[M+H-H2O]1+	
646.541	DG(37:05)_[M+NH4]1+	
633.582	DG(38:01)_[M+H-H2O]1+	
631.566	DG(38:02)_[M+H-H2O]1+	
627.535	DG(38:04)_[M+H-H2O]1+	
662.572	DG(38:04)_[M+NH4]1+	
623.504	DG(38:06)_[M+H-H2O]1+	
621.488	DG(38:07)_[M+H-H2O]1+	
661.613	DG(40:01)_[M+H-H2O]1+	
659.598	DG(40:02)_[M+H-H2O]1+	
692.619	DG(40:03)_[M+NH4]1+	
655.566	DG(40:04)_[M+H-H2O]1+	
651.535	DG(40:06)_[M+H-H2O]1+	
686.572	DG(40:06)_[M+NH4]1+	
649.520	DG(40:07)_[M+H-H2O]1+	
647.504	DG(40:08)_[M+H-H2O]1+	
677.645	DG(41:00)_[M+H-H2O]1+	
712.681	DG(41:00)_[M+NH4]1+	
675.629	DG(41:01)_[M+H-H2O]1+	
689.645	DG(42:01)_[M+H-H2O]1+	
687.629	DG(42:02)_[M+H-H2O]1+	
683.598	DG(42:04)_[M+H-H2O]1+	
718.634	DG(42:04)_[M+NH4]1+	
677.550	DG(42:07)_[M+H-H2O]1+	
712.588	DG(42:07)_[M+NH4]1+	
675.535	DG(42:08)_[M+H-H2O]1+	
671.504	DG(42:10)_[M+H-H2O]1+	
730.634	DG(43:05)_[M+NH4]1+	
754.728	DG(44:00)_[M+NH4]1+	
709.613	DG(44:05)_[M+H-H2O]1+	
705.582	DG(44:07)_[M+H-H2O]1+	
740.619	DG(44:07)_[M+NH4]1+	
703.566	DG(44:08)_[M+H-H2O]1+	SM(34:01)
699.535	DG(44:10)_[M+H-H2O]1+	
695.503	DG(44:12)_[M+H-H2O]1+	
735.629	DG(46:06)_[M+H-H2O]1+	
733.613	DG(46:07)_[M+H-H2O]1+	

468.309	LPC(14:00)		
496.340	LPC(16:00)		
494.324	LPC(16:01)	LPE(19:01)	
510.356	LPC(17:00)		
524.372	LPC(18:00)	PC-O(18:00)	
522.356	LPC(18:01)	PC-O(18:01)	PC-P(18:00)
520.340	LPC(18:02)	PC-O(18:02)	PC-P(18:01)
568.340	LPC(22:06)		
482.361	LPC-2O(16:00)		
508.376	LPC-O(18:01)		
506.361	LPC-O(18:02)	LPC-P(18:01)	
478.330	LPC-P(16:01)		
454.293	LPE(16:00)	LPC(13:00)	
482.324	LPE(18:00)	LPC(15:00)	
480.309	LPE(18:01)	LPC(15:01)	
538.387	LPE(22:00)	PC-O(19:00)	
526.293	LPE(22:06)		
438.298	LPE-P(16:00)		
541.350	LPG(20:00)		
556.361	LPG(20:01)_[M+NH4]1+		
531.272	LPG(20:05)	LPG(18:02)_[M+Na]1+	
555.366	LPG(21:00)		
573.303	LPI(16:00)		
600.314	LPI(17:02)_[M+NH4]1+		
618.361	LPI(18:00)_[M+NH4]1+		
630.398	LPI-P(20:00)_[M+NH4]1+		
570.280	LPS(20:03)_[M+Na]1+	LPS(22:06)	
566.249	LPS(20:05)_[M+Na]1+		
398.327	MG(20:03)_[M+NH4]1+		
396.311	MG(20:04)_[M+NH4]1+		
661.384	PA(32:05)_[M+Na]1+		
697.478	PA(34:01)_[M+Na]1+		
735.494	PA(37:03)_[M+Na]1+		
731.462	PA(37:05)_[M+Na]1+		
678.507	PC(28:00)	PE(31:00)	
706.539	PC(30:00)	PE(33:00)	
734.570	PC(32:00)	PE(35:00)	
730.539	PC(32:02)	PE(35:02)	
760.586	PC(34:01)	PE(37:01)	
758.570	PC(34:02)	PE(37:02)	
756.554	PC(34:03)	PE(37:03)	
754.539	PC(34:04)	PE(37:04)	
788.617	PC(36:01)	PE(39:01)	
786.601	PC(36:02)	PE(39:02)	
784.586	PC(36:03)	PE(39:03)	
782.570	PC(36:04)	PE(39:04)	
780.554	PC(36:05)	PE(39:05)	
776.523	PC(36:07)	PE(39:07)	
816.648	PC(38:01)	PE(41:01)	
814.633	PC(38:02)	PE(41:02)	
812.617	PC(38:03)	PE(41:03)	
810.601	PC(38:04)	PE(41:04)	
808.586	PC(38:05)	PE(41:05)	
806.570	PC(38:06)	PE(41:06)	
804.554	PC(38:07)		
802.538	PC(38:08)		
800.523	PC(38:09)		
846.695	PC(40:00)	PE(43:00)	
844.680	PC(40:01)	PE(43:01)	
842.664	PC(40:02)	PE(43:02)	
840.648	PC(40:03)	PE(43:03)	
838.633	PC(40:04)	PE(43:04)	
834.601	PC(40:06)	PE(43:06)	

832.586	PC(40:07)	PE(43:07)	
830.570	PC(40:08)	PE(43:08)	
828.554	PC(40:09)	PE(43:09)	
872.711	PC(42:01)	PE(45:01)	
870.695	PC(42:02)	PE(45:02)	
868.679	PC(42:03)	PE(45:03)	
866.663	PC(42:04)	PE(45:04)	
864.648	PC(42:05)	PE(45:05)	
860.616	PC(42:07)	PE(45:07)	
858.601	PC(42:08)	PE(45:08)	
852.554	PC(42:11)	PE(45:11)	
902.758	PC(44:00)	PE(47:00)	
900.742	PC(44:01)	PE(47:01)	
650.548	PC-2O(28:00)		
496.340	LPC(16:00)	LPE(19:00)	
522.355	LPC(18:01)	LPE(21:01)	
550.387	LPC(20:01)	LPE(23:01)	
664.528	PC-O(28:00)		
654.450	PC-O(28:05)	PC-P(28:04)	
720.591	PC-O(32:00)		
718.575	PC-O(32:01)	PC-P(32:00)	
730.575	PC-O(33:02)	PC-P(33:01)	
748.622	PC-O(34:00)		
746.606	PC-O(34:01)	PC-P(34:00)	
744.590	PC-O(34:02)	PE-O(37:02)	
776.653	PC-O(36:00)		
774.638	PC-O(36:01)	PE-P(39:00)	
772.622	PC-O(36:02)	PE-P(39:01)	TG_oxid(44:06)
766.575	PC-O(36:05)	PC-P(36:04)	
804.685	PC-O(38:00)		
800.653	PC-O(38:02)	PE-P(41:01)	
830.700	PC-O(40:01)	PC-P(40:00)	
828.684	PC-O(40:02)	PC-P(40:01)	
826.669	PC-O(40:03)	PC-P(40:02)	
822.637	PC-O(40:05)	PC-P(40:04)	
814.575	PC-O(40:09)	PC-P(40:08)	
852.684	PC-O(42:04)	PC-P(42:03)	
850.669	PC-O(42:05)	PC-P(42:04)	
838.575	PC-O(42:11)	PC-P(42:10)	
906.732	PC-O(46:05)	PC-P(46:03)	
902.700	PC-O(46:07)	PC-P(46:06)	
942.826	PC-O(48:01)		
816.591	PC-O(40:08)	PC-P(40:07)	
836.653	PC-O(41:05)	PC-P(41:04)	
852.685	PC-O(42:04)	PC-P(42:03)	
680.429	PE(32:06)		
696.460	PE(33:05)	PA(35:06)_[M+NH4]1+	
720.554	PE(34:00)	PC(31:00)	
718.539	PE(34:01)	PC(31:01)	
716.523	PE(34:02)	PC(31:02)	
748.586	PE(36:00)	PC(33:00)	
744.554	PE(36:02)	PC(33:02)	
742.539	PE(36:03)	PC(33:03)	
736.491	PE(36:06)	PC(33:06)	PA(38:07)_[M+NH4]1+
774.601	PE(38:01)	PC(35:01)	
772.586	PE(38:02)	PC(35:02)	
764.523	PE(38:06)	PC(35:06)	
804.648	PE(40:00)	PC(37:00)	PA(42:01)_[M+NH4]1+
802.633	PE(40:01)	PC(37:01)	
800.617	PE(40:02)	PC(37:02)	
798.601	PE(40:03)	PC(37:03)	
796.586	PE(40:04)	PC(37:04)	
792.554	PE(40:06)	PC(37:06)	

830.664	PE(42:01)	PC(39:01)	
828.648	PE(42:02)	PC(39:02)	
822.601	PE(42:05)	PC(39:05)	
814.539	PE(42:09)	PE(39:09)	
858.695	PE(44:01)	PC(41:01)	
886.727	PE(46:01)	PC(43:01)	
878.664	PE(46:05)	PC(43:05)	
676.528	PE-O(32:01)	PC-P(29:00)	
700.528	PE-O(34:03)	PC-O(31:03)	
728.559	PE-O(36:03)	PC-O(33:03)	
726.543	PE-O(36:04)	PC-O(33:04)	
756.590	PE-O(38:03)	PC-O(35:01)	PC-P(35:02)
754.575	PE-O(38:04)	PC-O(35:04)	
752.559	PE-O(38:05)	PC-O(35:05)	
786.637	PE-O(40:02)	PC-O(37:02)	
784.622	PE-O(40:03)	PC-P(37:02)	
782.606	PE-O(40:04)	PC-O(37:04)	
780.590	PE-O(40:05)		
778.575	PE-O(40:06)		
810.637	PE-O(42:04)		
806.606	PE-O(42:06)		
674.512	PE-P(32:01)		
748.528	PE-P(38:06)		
774.543	PE-P(40:07)		
611.392	PG(24:00)		
642.434	PG(25:00)_[M+NH4]1+		
656.450	PG(26:00)_[M+NH4]1+		
653.439	PG(27:00)		
698.497	PG(29:00)_[M+NH4]1+		
712.512	PG(30:00)_[M+NH4]1+		
743.483	PG(32:01)_[M+Na]1+	PG(34:04)	
749.533	PG(34:01)		
771.515	PG(34:01)_[M+Na]1+	PG(36:04)	
747.517	PG(34:02)		
779.580	PG(36:00)		
801.562	PG(36:00)_[M+Na]1+		
799.546	PG(36:01)_[M+Na]1+	PG(38:04)	
775.548	PG(36:02)		
797.530	PG(36:02)_[M+Na]1+		
807.611	PG(38:00)		
801.564	PG(38:03)		
797.533	PG(38:05)		
817.499	PG(38:06)_[M+Na]1+		
791.486	PG(38:08)		
831.515	PG(39:06)_[M+Na]1+		
829.499	PG(39:07)_[M+Na]1+		
833.627	PG(40:01)		
845.530	PG(40:06)_[M+Na]1+	PG(42:09)	
843.515	PG(40:07)_[M+Na]1+		
866.685	PG(41:00)_[M+NH4]1+		
857.530	PG(41:07)_[M+Na]1+		
709.538	PG-O(32:00)		
768.611	PG-O(35:00)_[M+NH4]1+		
779.616	PG-O(37:00)	TG(45:04)_[M+Na]1+	
777.600	PG-O(37:01)	TG(45:05)_[M+Na]1+	
791.616	PG-O(38:01)	TG(46:05)_[M+Na]1+	
835.679	PG-O(41:00)	TG(49:04)_[M+Na]1+	
852.705	PG-O(41:00)_[M+NH4]1+		
859.582	PG-O(42:06)_[M+Na]1+		
725.424	PI(26:01)		
755.471	PI(28:00)		
768.466	PI(28:02)_[M+NH4]1+		
769.486	PI(29:00)		

784.497	PI(29:01)_[M+NH4]1+	
800.528	PI(30:00)_[M+NH4]1+	
795.502	PI(31:01)	
824.528	PI(32:02)_[M+NH4]1+	
820.497	PI(32:04)_[M+NH4]1+	
821.518	PI(33:02)	
838.544	PI(33:02)_[M+NH4]1+	
832.497	PI(33:05)_[M+NH4]1+	
856.591	PI(34:00)_[M+NH4]1+	PC(42:09)
837.549	PI(34:01)	
859.531	PI(34:01)_[M+Na]1+	PI(36:04)
835.533	PI(34:02)	
857.515	PI(34:02)_[M+Na]1+	
831.502	PI(34:04)	
829.486	PI(34:05)	
866.575	PI(35:02)_[M+NH4]1+	
845.518	PI(35:04)	PI(33:01)_[M+Na]1+
862.544	PI(35:04)_[M+NH4]1+	
860.528	PI(35:05)_[M+NH4]1+	
858.513	PI(35:06)_[M+NH4]1+	
867.596	PI(36:00)	
887.562	PI(36:01)_[M+Na]1+	PI(38:04)
882.607	PI(36:01)_[M+NH4]1+	
885.546	PI(36:02)_[M+Na]1+	PI(38:05)
878.569	PI(36:03)_[M+NH4]1+	PC(44:12)
881.515	PI(36:04)_[M+Na]1+	PI(38:07)
857.518	PI(36:05)	
898.638	PI(37:00)_[M+NH4]1+	
879.596	PI(37:01)	
873.549	PI(37:04)	
871.533	PI(37:05)	
888.560	PI(37:05)_[M+NH4]1+	
909.546	PI(38:04)_[M+Na]1+	PI(40:07)
904.591	PI(38:04)_[M+NH4]1+	
883.533	PI(38:06)	
905.611	PI(39:02)	
814.580	PI-O(32:00)_[M+NH4]1+	
817.520	PI-O(32:01)_[M+Na]1+	
824.565	PI-O(33:02)_[M+NH4]1+	
819.538	PI-O(34:03)	
841.520	PI-O(34:03)_[M+Na]1+	PI-P(36:05)
817.523	PI-O(34:04)	
867.536	PI-O(36:04)_[M+Na]1+	PI-P(38:06)
860.565	PI-O(36:05)_[M+NH4]1+	
867.632	PI-O(37:00)	
893.648	PI-O(39:01)	
775.473	PI-P(29:00)_[M+Na]1+	
787.473	PI-P(30:01)_[M+Na]1+	
779.507	PI-P(31:01)	
796.533	PI-P(31:01)_[M+NH4]1+	
815.507	PI-P(34:04)	
858.549	PI-P(36:05)_[M+NH4]1+	
861.585	PI-P(37:02)	
646.369	PS(24:00)_[M+Na]1+	
650.403	PS(26:01)	
722.497	PS(31:00)	PG(31:02)_[M+NH4]1+
718.465	PS(31:02)	PG(31:04)_[M+NH4]1+
746.497	PS(33:02)	PG(33:04)_[M+NH4]1+
784.510	PS(34:01)	
794.497	PS(35:03)	PS(37:06)
792.479	PS(35:04)	
768.481	PS(35:05)	
784.512	PS(36:04)	PG(36:06)_[M+NH4]1+

780.479	PS(36:06)	PG(36:08)_[M+NH4]1+	PS(34:03)_[M+Na]1+
802.559	PS(37:02)	PG(37:04)_[M+NH4]1+	
800.544	PS(37:03)	PG(37:05)_[M+NH4]1+	
822.526	PS(37:03)		
820.510	PS(37:04)		
818.591	PS(38:01)	PG(38:03)_[M+NH4]1+	
812.544	PS(38:04)	PG(38:06)_[M+NH4]1+	PS(36:01)_[M+Na]1+
806.497	PS(38:07)	PG(38:09)_[M+NH4]1+	PS(36:04)_[M+Na]1+
822.528	PS(39:06)	PG(39:08)_[M+NH4]1+	
844.510	PS(39:06)		
820.512	PS(39:07)		
842.588	PS(40:03)	PG(40:05)_[M+NH4]1+	PS(38:00)_[M+Na]1+
862.559	PS(40:04)_[M+Na]1+	PS(42:07)	PG(42:09)_[M+NH4]1+
860.541	PS(40:05)_[M+Na]1+	PS(42:08)	PG(42:10)_[M+NH4]1+
836.544	PS(40:06)	PG(40:08)_[M+NH4]1+	PS(38:03)_[M+Na]1+
834.528	PS(40:07)	PG(40:09)_[M+NH4]1+	PS(38:04)_[M+Na]1+
856.510	PS(40:07)_[M+Na]1+	PS(42:10)	
832.512	PS(40:08)	PG(40:10)_[M+NH4]1+	PS(38:05)_[M+Na]1+
830.497	PS(40:09)		
858.622	PS(41:02)	PG(41:04)_[M+NH4]1+	
856.606	PS(41:03)	PG(41:05)_[M+NH4]1+	
848.544	PS(41:07)	PS(39:04)_[M+Na]1+	
872.638	PS(42:02)	PG(42:04)_[M+NH4]1+	
894.620	PS(42:02)		
880.510	PS(42:09)		
876.479	PS(42:11)		
908.635	PS(43:02)		
922.651	PS(44:02)		
888.573	PS(44:08)	PG(44:10)_[M+NH4]1+	PS(42:05)_[M+Na]1+
910.557	PS(44:08)_[M+Na]1+	PI(39:08)_[M+NH4]1+	
884.544	PS(44:10)	PG(44:12)_[M+NH4]1+	PS(42:07)_[M+Na]1+
816.515	PS-O(38:06)_[M+Na]1+		
840.609	PS-O(39:01)_[M+Na]1+		
842.531	PS-P(40:06)_[M+Na]1+		
687.544	SM(33:02)		
701.559	SM(34:02)		
719.606	SM(35:00)		
731.606	SM(36:01)		
729.591	SM(36:02)		
725.559	SM(36:04)		
759.638	SM(38:01)		
757.622	SM(38:02)		
753.591	SM(38:04)		
789.684	SM(40:00)		
787.669	SM(40:01)		
785.653	SM(40:02)		
803.700	SM(41:00)		
801.684	SM(41:00)		
817.716	SM(42:00)		
815.700	SM(42:01)		
813.684	SM(42:02)		
811.669	SM(42:03)		
825.685	SM(43:03)		
823.669	SM(43:04)		
845.747	SM(44:00)		
837.685	SM(44:04)		
835.669	SM(44:05)		
873.778	SM(46:00)		
871.763	SM(46:01)		
899.794	SM(48:01)		
614.536	TG(33:00)		
642.567	TG(35:00)		
640.552	TG(35:01)		

661.538	TG(36:00)_[M+Na]1+		
675.553	TG(37:00)_[M+Na]1+		
689.569	TG(38:00)_[M+Na]1+	SM(33:01)	
678.567	TG(38:03)		
674.536	TG(38:05)		
717.600	TG(40:00)_[M+Na]1+	SM(35:01)	
712.645	TG(40:00)_[M+NH4]1+		
727.585	TG(41:02)_[M+Na]1+		
740.676	TG(42:00)_[M+NH4]1+		
743.616	TG(42:01)_[M+Na]1+		
741.600	TG(42:02)_[M+Na]1+		
736.645	TG(42:02)_[M+NH4]1+		
732.614	TG(42:04)_[M+NH4]1+		
752.676	TG(43:01)_[M+NH4]1+		
755.616	TG(43:02)_[M+Na]1+		
748.645	TG(43:03)_[M+NH4]1+		
764.676	TG(44:02)_[M+NH4]1+		
762.661	TG(44:03)_[M+NH4]1+		
783.647	TG(45:02)_[M+Na]1+		
776.677	TG(45:03)		
781.632	TG(45:03)_[M+Na]1+		
774.661	TG(45:04)_[M+NH4]1+		
796.739	TG(46:00)_[M+NH4]1+		
799.679	TG(46:01)_[M+Na]1+	SM(41:01)	
794.723	TG(46:01)_[M+NH4]1+		
797.663	TG(46:02)_[M+Na]1+		
792.708	TG(46:02)_[M+NH4]1+		
790.692	TG(46:03)_[M+NH4]1+		
788.676	TG(46:04)_[M+NH4]1+		
786.661	TG(46:05)_[M+NH4]1+		
784.645	TG(46:06)_[M+NH4]1+		
809.663	TG(47:03)_[M+Na]1+		
807.647	TG(47:04)_[M+Na]1+	SM(42:05)	PG-O(39:00)
829.726	TG(48:00)_[M+Na]1+		
827.710	TG(48:01)_[M+Na]1+		
822.755	TG(48:01)_[M+NH4]1+		
820.739	TG(48:02)_[M+NH4]1+		
818.723	TG(48:03)_[M+NH4]1+		
816.708	TG(48:04)_[M+NH4]1+		
814.692	TG(48:05)_[M+NH4]1+		
812.676	TG(48:06)_[M+NH4]1+		
841.726	TG(49:01)_[M+Na]1+		
836.770	TG(49:01)_[M+NH4]1+		
837.694	TG(49:03)_[M+Na]1+		
832.739	TG(49:03)_[M+NH4]1+		
830.723	TG(49:04)_[M+NH4]1+		
833.663	TG(49:05)_[M+Na]1+	SM(44:06)	PG-P(41:00)
831.647	TG(49:06)_[M+Na]1+		
857.757	TG(50:00)_[M+Na]1+		
855.741	TG(50:01)_[M+Na]1+		
850.786	TG(50:01)_[M+NH4]1+		
848.770	TG(50:02)_[M+NH4]1+		
846.755	TG(50:03)_[M+NH4]1+		
844.739	TG(50:04)_[M+NH4]1+		
840.708	TG(50:06)_[M+NH4]1+		
838.692	TG(50:07)_[M+NH4]1+	PG-O(40:00)_[M+NH4]1+	
832.645	TG(50:10)_[M+NH4]1+		
866.817	TG(51:00)_[M+NH4]1+		
864.802	TG(51:01)_[M+NH4]1+		
858.755	TG(51:04)_[M+NH4]1+		
883.773	TG(52:01)_[M+Na]1+		
878.817	TG(52:01)_[M+NH4]1+		
881.757	TG(52:02)_[M+Na]1+		

876.802	TG(52:02)_[M+NH4]1+
879.741	TG(52:03)_[M+Na]1+
874.786	TG(52:03)_[M+NH4]1+
872.770	TG(52:04)_[M+NH4]1+
870.755	TG(52:05)_[M+NH4]1+
868.739	TG(52:06)_[M+NH4]1+
890.817	TG(53:02)_[M+NH4]1+
888.802	TG(53:03)_[M+NH4]1+
884.770	TG(53:05)_[M+NH4]1+
880.739	TG(53:07)_[M+NH4]1+
874.692	TG(53:10)_[M+NH4]1+
911.804	TG(54:01)_[M+Na]1+
906.848	TG(54:01)_[M+NH4]1+
909.788	TG(54:02)_[M+Na]1+
904.833	TG(54:02)_[M+NH4]1+
902.817	TG(54:03)_[M+NH4]1+
900.802	TG(54:04)_[M+NH4]1+
903.741	TG(54:05)_[M+Na]1+
898.786	TG(54:05)_[M+NH4]1+
896.770	TG(54:06)_[M+NH4]1+
894.755	TG(54:07)_[M+NH4]1+
892.739	TG(54:08)_[M+NH4]1+
891.647	TG(54:11)_[M+Na]1+
918.848	TG(55:02)_[M+NH4]1+
916.833	TG(55:03)_[M+NH4]1+
914.817	TG(55:04)_[M+NH4]1+
912.802	TG(55:05)_[M+NH4]1+
908.770	TG(55:07)_[M+NH4]1+
906.755	TG(55:08)_[M+NH4]1+
902.723	TG(55:10)_[M+NH4]1+
934.880	TG(56:01)_[M+NH4]1+
930.848	TG(56:03)_[M+NH4]1+
928.833	TG(56:04)_[M+NH4]1+
926.817	TG(56:05)_[M+NH4]1+
924.802	TG(56:06)_[M+NH4]1+
922.786	TG(56:07)_[M+NH4]1+
920.770	TG(56:08)_[M+NH4]1+
918.755	TG(56:09)_[M+NH4]1+
961.820	TG(58:04)_[M+Na]1+
956.864	TG(58:04)_[M+NH4]1+
954.848	TG(58:05)_[M+NH4]1+
952.833	TG(58:06)_[M+NH4]1+
948.802	TG(58:08)_[M+NH4]1+
946.786	TG(58:09)_[M+NH4]1+
944.770	TG(58:10)_[M+NH4]1+
986.911	TG(60:03)_[M+NH4]1+
989.851	TG(60:04)_[M+Na]1+
982.880	TG(60:05)_[M+NH4]1+
976.833	TG(60:08)_[M+NH4]1+
974.817	TG(60:09)_[M+NH4]1+
990.848	TG(61:08)_[M+NH4]1+
1025.757	TG(64:14)_[M+Na]1+
668.546	TG_oxid(36:02)
686.593	TG_oxid(37:00)
696.577	TG_oxid(38:02)
722.593	TG_oxid(40:03)
740.640	TG_oxid(41:01)
738.624	TG_oxid(41:02)
750.624	TG_oxid(42:03)
748.609	TG_oxid(42:04)
766.655	TG_oxid(43:02)
764.640	TG_oxid(43:03)
780.671	TG_oxid(44:02)

776.640	TG_oxid(44:04)
798.718	TG_oxid(45:00)
794.687	TG_oxid(45:02)
790.655	TG_oxid(45:04)
808.702	TG_oxid(46:02)
806.687	TG_oxid(46:03)
804.671	TG_oxid(46:04)
840.765	TG_oxid(48:00)
830.687	TG_oxid(48:05)
862.749	TG_oxid(50:03)
892.796	TG_oxid(52:02)
884.734	TG_oxid(52:06)
908.828	TG_oxid(53:01)
920.828	TG_oxid(54:02)
918.812	TG_oxid(54:03)
916.796	TG_oxid(54:04)
946.843	TG_oxid(56:03)
958.843	TG_oxid(57:04)
954.812	TG_oxid(57:06)
950.781	TG_oxid(57:08)
944.734	TG_oxid(57:11)
1006.749	TG_oxid(62:15)
1034.781	TG_oxid(64:15)
