# Supplementary material for: Lipid Traffic Analysis reveals the impact of high paternal carbohydrate intake on offsprings’ lipid metabolism
Source: Commun Biol. 2021 Feb 5;4:163. doi: 10.1038/s42003-021-01686-1 (PMC7864968; doi:10.1038/s42003-021-01686-1)
Supplement: Supplementary file 9 — Reporting Summary [file 42003_2021_1686_MOESM9_ESM.pdf]

Corresponding author(s): Dr Samuel Furse, sf615@cam.ac.uk, samuel@samuelfurse.com

Last updated by author(s): Dec 5, 2020

Reporting Summary

Nature Research wishes to improve the reproducibility of the work that we publish. This form provides structure for consistency and transparency in reporting. For further information on Nature Research policies, see our Editorial Policies and the Editorial Policy Checklist.

Statistics

For all statistical analyses, confirm that the following items are present in the figure legend, table legend, main text, or Methods section.

n/a	Confirmed	
☐	☒	The exact sample size (*n*) for each experimental group/condition, given as a discrete number and unit of measurement
☐	☒	A statement on whether measurements were taken from distinct samples or whether the same sample was measured repeatedly
☐	☒	The statistical test(s) used AND whether they are one- or two-sided<br>*Only common tests should be described solely by name; describe more complex techniques in the Methods section.*
☐	☒	A description of all covariates tested
☐	☒	A description of any assumptions or corrections, such as tests of normality and adjustment for multiple comparisons
☐	☒	A full description of the statistical parameters including central tendency (e.g. means) or other basic estimates (e.g. regression coefficient) AND variation (e.g. standard deviation) or associated estimates of uncertainty (e.g. confidence intervals)
☐	☒	For null hypothesis testing, the test statistic (e.g. *F*, *t*, *r*) with confidence intervals, effect sizes, degrees of freedom and *P* value noted<br>*Give P values as exact values whenever suitable.*
☒	☐	For Bayesian analysis, information on the choice of priors and Markov chain Monte Carlo settings
☒	☐	For hierarchical and complex designs, identification of the appropriate level for tests and full reporting of outcomes
☒	☐	Estimates of effect sizes (e.g. Cohen's *d*, Pearson's *r*), indicating how they were calculated

Our web collection on statistics for biologists contains articles on many of the points above.

Software and code

Policy information about availability of computer code

Data collection	Raw high-resolution mass-spectrometry data were processed using XCMS (www.bioconductor.org) and Peakpicker v 2.0 (an in-house R script). 31P NMR data was processed using TopSpin 4.0.7. OriginLab 2018 or Excel 2016 were used for calculations and preparation of figures.
Data analysis	New code was developed for the purpose of a spatial network analysis, this code (Lipid Traffic Analysis v1.0) is given with the manuscript as is a description of how it works and what was done with it.

For manuscripts utilizing custom algorithms or software that are central to the research but not yet described in published literature, software must be made available to editors and reviewers. We strongly encourage code deposition in a community repository (e.g. GitHub). See the Nature Research guidelines for submitting code & software for further information.

Data

Policy information about availability of data

All manuscripts must include a data availability statement. This statement should provide the following information, where applicable:

- Accession codes, unique identifiers, or web links for publicly available datasets
- A list of figures that have associated raw data
- A description of any restrictions on data availability

All data and code used in the present study are provided as Electronic Supplementary Material.

Field-specific reporting

Please select the one below that is the best fit for your research. If you are not sure, read the appropriate sections before making your selection.

[x] Life sciences [ ] Behavioural & social sciences [ ] Ecological, evolutionary & environmental sciences

For a reference copy of the document with all sections, see nature.com/documents/nr-reporting-summary-flat.pdf

Life sciences study design

All studies must disclose on these points even when the disclosure is negative.

Sample size	This was a mouse study with group sizes of up to 8.
Data exclusions	Variables identified in the switch analysis were not identified in the abundance analysis.
Replication	The number of sires used was based on prior power calculations using our previously published data to ensure replication of model phenotypic effects. Tissues were sampled from across several litters (and therefore several fathers) which all showed similar phenotypic changes in growth and metabolic status. Finally, these data are in line with our previous published studies using this model.
Randomization	Mice were randomly assigned to dietary groups after acclimatisation to the facility. Mass spectrometry samples were run in random order.
Blinding	Sample names afforded partial blinding, though as around 1100 mass spectrometry samples were run in a high throughput manner in a random order, further blinding was not considered necessary.

Reporting for specific materials, systems and methods

We require information from authors about some types of materials, experimental systems and methods used in many studies. Here, indicate whether each material, system or method listed is relevant to your study. If you are not sure if a list item applies to your research, read the appropriate section before selecting a response.

Materials & experimental systems

n/a	Involved in the study	
[x]	[ ]	Antibodies
[x]	[ ]	Eukaryotic cell lines
[x]	[ ]	Palaeontology and archaeology
[ ]	[x]	Animals and other organisms
[x]	[ ]	Human research participants
[x]	[ ]	Clinical data
[x]	[ ]	Dual use research of concern

Methods

n/a	Involved in the study	
[x]	[ ]	ChIP-seq
[x]	[ ]	Flow cytometry
[x]	[ ]	MRI-based neuroimaging

Animals and other organisms

Policy information about studies involving animals; ARRIVE guidelines recommended for reporting animal research

Laboratory animals	C57BL/6 mice
Wild animals	No wild animals
Field-collected samples	No samples collected from the field
Ethics oversight	All procedures were conducted in accordance with the UK Home Office Animal (Scientific Procedures) Act 1986 and local ethics committees at Aston University.

Note that full information on the approval of the study protocol must also be provided in the manuscript.
